# Social inequalities in vaccine coverage and their effects on epidemic spreading

**DOI:** 10.1371/journal.pcbi.1013585

**Published:** 2025-10-13

**Authors:** Adriana Manna, Márton Karsai, Nicola Perra

**Affiliations:** 1 Department of Network and Data Science, Central European University, Vienna, Austria; 2 National Laboratory for Health Security, HUN-REN Alfréd Rényi Institute of Mathematics, Budapest, Hungary; 3 School of Mathematical Sciences, Queen Mary University of London, London, United Kingdom; 4 The Alan Turing Institute, London, United Kingdom; University of Washington, UNITED STATES OF AMERICA

## Abstract

Vaccinations are fundamental public health interventions. Yet, inequalities in vaccine uptake across socioeconomic groups can significantly undermine their impact. Moreover, heterogeneities in vaccination coverage across socioeconomic strata are typically neglected by epidemic models and considered, if at all, only at posteriori. This limitation reduces their ability to predict and assess the effectiveness of vaccination campaigns. Here, we study the impact of socioeconomic inequalities in vaccination uptake on disease burden, measured as attack rate. We consider a modeling framework based on generalized contact matrices that extend traditional age-stratified approaches to incorporate socioeconomic status (SES) variables. We simulate epidemic dynamics under two scenarios. In the first, vaccination campaigns are concurrent with epidemics. In the second, instead, vaccinations are completed before the onset of infection waves. By using both synthetic and empirical generalized contact matrices, we find that inequalities in vaccine uptake can lead to non-linear effects on disease outcomes and exacerbate disease burden in disadvantaged groups of the population. We demonstrate that simpler models ignoring SES heterogeneity produce incomplete or biased predictions of attack rates. Additionally, we show how inequalities in vaccine coverage interact with non-pharmaceutical interventions (NPIs), compounding differences across subgroups. Overall, our findings highlight the importance of integrating SES dimensions, alongside age, into epidemic models to inform more equitable and effective public health interventions and vaccination strategies.

## Author summary

We investigate how social inequalities in vaccination uptake influence epidemic outcomes. To this end, we adopt a generalized modeling framework to incorporate socioeconomic status (SES) with age in the description of the population and their contacts. We simulate epidemic scenarios under different vaccination distributions and timings. Our findings reveal that inequalities in vaccine coverage can lead to disproportionate disease burdens among disadvantaged groups, especially when compounded by possible heterogeneities in the adoption of non-pharmaceutical interventions (NPIs). By analyzing both synthetic and real-world data from Hungary, we demonstrate that traditional age-structured epidemic models underestimate the effects of SES inequalities, potentially leading to biased predictions and suboptimal public health responses. Our study highlights the critical importance of integrating social inequalities in vaccination into epidemic models to ensure accurate descriptions and equitable interventions. These results provide actionable insights for policymakers and public health officials to improve population health outcomes.

## Introduction

Vaccination is one of the most effective public health interventions for controlling infectious diseases, protecting individuals and communities [1]. Besides their efficacy to different endpoints (e.g., infection, death), vaccine access and uptake are key factors shaping the impact on disease burden. Vaccines are designed to be accessible to all, but limited stockpiles often raise the issue of optimal distribution [2,3]. Common protocols prioritise essential workers, the elderly, children, or individuals with underlying health conditions. Social status should not determine access and yet, as clearly observed during the COVID-19 Pandemic, vaccination access is shaped by significant inequalities across and within countries [4–10]. Indeed, studies have shown that individuals from lower socioeconomic backgrounds are less likely to be vaccinated against COVID-19 [10–13]. For example, disparities have been observed in Florida’s initial vaccine rollout [14], where lower-income and minority communities faced significant obstacles in accessing vaccines. Geographic and ethnic disparities also play a crucial role, as seen in Hungary, where socioeconomic deprivation has been found to be correlated with lower vaccination coverage [13,15,16]. In the Greater Manchester area in the UK, COVID-19 vaccination rates have been lower in 15 of the 16 minority ethnic groups [17].

The correlation between socioeconomic status (SES) and vaccination uptake varies across different illnesses [18–20], but individuals experiencing lower SES exhibit lower vaccination rates due to barriers like limited access to healthcare, but also exposure to (mis)information, and vaccine hesitancy [19,21]. Indeed, social and behavioural factors emergent from interactions and (mis)information exposure play an important role in vaccine uptake [19,22,23]. Studies in France have highlighted higher COVID-19 vaccine hesitancy among groups experiencing lower SES, driven by concerns over vaccine safety and distrust in government health measures [24,25]. Overall, all these findings remain consistent regardless of age, indicating that socioeconomic factors play a crucial role in vaccine hesitancy and access across all age groups [26,27].

Additionally, for any given uptake and coverage level, the impact of a vaccination campaign is closely tied to its timing and speed relative to the progression of the epidemic [10]. A campaign, that begins only after a large portion of the population has already been infected, will inevitably have a much smaller effect compared to one initiated earlier. During the COVID-19 Pandemic, vaccinations started in December 2020 (in the Global North) [28,29]. At that time many countries were facing a rise in infections induced by the winter seasonality and by the spread of a more transmissible variant (i.e., Alpha). Furthermore, doses were a scarce resource and countries had to deal with tremendous logistical challenges for their distribution. As a result, the initial vaccination rates were far from optimal. Hence, the beginning of COVID-19 vaccinations is a clear example of a campaign that starts amid an epidemic and finds a large majority of the population susceptible. Non-pharmaceutical interventions (NPIs) have been critical to support these complex phases. However, NPIs have also been associated with socioeconomic inequalities. Indeed, the ability to self-isolate, work from home, and avoid crowded places varied significantly across different socioeconomic groups [9,30–40]. Consequently, disadvantaged parts of the population, including specific ethnic groups [41,42], suffered from compounding inequalities. On one side, they had limited access to vaccines, as well as other medications [43]. On the other, they were more exposed to infections. It is important to notice how such health inequalities are not limited to COVID-19. On the contrary they are found across a wide range of diseases [20,44,45]. They have significant implications for public health and perpetuate the spread of vaccine-preventable diseases.

In this context, incorporating socioeconomic factors (among others) in epidemic models, thus going beyond the traditional age-wise stratification, is essential to quantify the impact of vaccinations [35,44–51]. However, the large majority of methods proposed so far fail to do so, potentially resulting in incomplete or biased predictions about the effectiveness of vaccination campaigns. We note a few exceptions. Larsen et al. used contact matrices stratified by SES, though not by age, to explore the link between vaccinations’ temporal trends and socioeconomic inequalities during the COVID-19 Pandemic in the USA [10]. The research highlights how the timing of the rollout impacts the disease’s burden more than both final coverage and disparities along SES dimensions. Alvarez-Zuzek et al. studied the link between SES and vaccine hesitancy via a spatial network model. Interestingly, they compared two social mechanisms that might be driving the spatial distribution of hesitancy [19]. They found that both social selection and social influence are able to reproduce the empirical spatial distributions of hesitancy in the USA. Zipfel et al. studied the impact of SES inequalities in the spread of influenza by using a contact network model [20]. They considered a data driven heterogeneity in vaccine coverage as a function of the SES (i.e., low VS high SES). The work confirms that individuals experiencing low SES are indeed disproportionately affected by the disease.

Here, we adopt and extend the epidemic modeling framework proposed in Ref [52] that features *generalized contact matrices*. The model allows accounting for the stratification of contacts across multiple dimensions such as age and SES [52]. In doing so, it provides a finer description of the population under investigation and allows to assess how disparities in vaccination coverage and NPIs adoption influence the overall epidemic outcome. To isolate the interplay between social contacts and vaccination uptake across subgroups, we consider both synthetic and real generalized contact matrices that stratify contacts according to age and one SES indicator. The age stratification is data-driven in both cases, while the stratification for the SES dimension is built either by using a simple model or from data collected in Hungary [53,54]. To account for the effect of vaccination timing, these analyses are developed in two distinct scenarios. The first simulates a situation where the vaccination campaign and the epidemic are concurrent. The second scenario instead simulates a situation in which the vaccination campaign begins before the epidemic, reaching a substantial portion of the population prior to the outbreak. This scenario is akin to the arrival of a new variant—like Omicron during the COVID-19 Pandemic—able to breach both natural and vaccine-induced immunity.

Our results emphasize the importance of models that explicitly account for socioeconomic disparities to accurately estimate the number of individuals that have been infected during an epidemic (i.e., attack rate) within specific subgroups of the population. Indeed, we demonstrate that, when disparities in vaccination uptake exist across subgroups, models that do not incorporate these dimensions fail to accurately predict the impact of the disease within these groups. Furthermore, we find that the unequal distribution of vaccines across socioeconomic groups might lead to non-linear effects on epidemic outcomes that, in general, cannot be estimated *a posteriori* from models that account just for the traditional age stratification. Finally, our findings show how disparities intensify when compounded by the interaction with NPIs.

Overall, our results underscore the importance of incorporating subgroup-specific factors into epidemic models to enhance the accuracy of predictions and, in turn, the effectiveness of public health interventions and vaccination distributions.

## Results

We consider a Susceptible-Exposed-Infectious-Recovered (SEIR) compartmental model incorporating multidimensional contact matrices with an additional dimension with three levels (i.e., subgroups), besides age [52]. We take into account eight age classes [0–5), [5–15), [15–30), [30–45), [45–60), [60–70), [70–80), [80+), and three SES: low, middle, and high. Specifically, we use *generalized contact matrices* **G** as multidimensional objects, whose elements ${𝐆}_{a,b}$ capture the contact rates between individuals in group **a** and **b**. Here, **a** = (*i*, *α*) and **b** = (*j*, *β*) are index vectors (i.e., tuples) representing individual’s membership to each category defined along age *i*, *j* and the second dimension α,β. The stratification for age is obtained from data [53,54], while the stratification for SES is either synthetic, obtained from a simple model, or measured from data. In the first case, we introduce assortativity by assuming that 60%, 50%, and 65% of the contacts in the first, second, and third SES category take place within each group. This distribution is inspired by observations in real data. Indeed, as shown in S1 Text (see Sect 9.1), in Hungary, individuals experiencing high and low SES are indeed the most assortative. We note, however, how assortativity generally decreases across SES, with age (see S1 Text for more details). For simplicity, when building synthetic generalised contact matrices we neglect this dependence. Interestingly, higher assortativity levels among individuals experiencing high SES have been found also in communication networks (via mobile phones) in Latin America [55]. We assume that activity levels are distributed heterogeneously, with 20%, 40%, and 40% of the total contacts allocated to the first, second, and third groups, respectively. As for assortativity, the distribution of activity is inspired by observations in Hungarian data [12]. Similar patterns have also been observed in Switzerland [56], where however the link between activity and SES is affected by other variables including education attainment. For simplicity, when building the synthetic generalised contact matrices we neglect the role of these modifying variables. Finally, the population distribution along the second dimension, i.e., SES, is based on data from Hungary, derived from the MASZK dataset. Following this data, we set an uneven population distribution with group sizes comprising 35%, 45%, and 20% of the total population. We note how we opted to consider only three categories in the second dimension to balance the complexity of the model with the capability to capture differences across sub-groups. Indeed, as shown in S1 Text, the number of free parameters needed to generate synthetic generalised contact matrices increases with the number of categories. The model we used to generate the synthetic population and mixing patterns along the second dimension follows Ref [52]. We report a summary of the model in S1 Text (see Sect 2).

The last part of the paper incorporates real data from Hungary to anchor the model in a specific and realistic setting. Here, we used empirically derived generalized contact matrices that reflect actual distributions of population structure, activity levels, and assortativity (see Sect 9 of S1 Text).

We model a vaccination rollout, where each day a fraction of the population receives a vaccine that reduces the probability of infection and death by $g_{1}=60\%$ and $g_{2}=80\%$ respectively [57]. Additionally, we assume that infected vaccinated individuals are 40% less likely to transmit the virus further. The daily allocation of vaccines to age groups is taken from data in Hungary reflecting the COVID-19 vaccination campaign (see S1 Text Sect 1.1). We study and compare four different vaccination uptake distributions (VD for short) across the SES subgroups. In the first case (*VD*1), doses are assigned proportionally to the share of the population of each subgroup. This strategy is a random distribution and acts as a baseline. We refer to the strategy as *random distribution*. In the second case (*VD*2), each subgroup receives an equal share, i.e., one-third of the vaccines. We refer to this strategy as *even distribution*. In the third case (*VD*3), doses are assigned targeting subgroups’ activity. We refer to this strategy as *activity based distribution*. Finally, in the fourth case (*VD*4), we consider real data from the Hungarian rollout. We follow the values reported in Ref [11]. We refer to this strategy as *realistic distribution*. We report the share of doses in each subgroup for all the strategies in S1 Text (see Table 3 in Sect 4.2.2). Under all vaccination distributions, we assume that each subgroup can be vaccinated up to 95%. We refer the reader to the Materials and methods Section and S1 Text for more details about the model and simulation setup.

Finally, as mentioned in the introduction, the interaction between the timing of a vaccination campaign and the progression of the epidemic plays a critical role and drives the disease burden [10]. To investigate their effect on the epidemic outcome, we focus on two simulation scenarios. In the first (*Scenario 1*), the epidemic and vaccination campaign start simultaneously. In the second scenario (*Scenario 2*), the vaccination campaign is completed before the epidemic begins. As mentioned above, this scenario is inspired by what we experienced with the emergence of the Omicron variant during the COVID-19 Pandemic. Indeed, Omicron was able to breach both natural and vaccine-induced immunity [58–60]. The variant spread when the Global North largely completed the rollout of two doses and was distributing boosters [60]. In both scenarios, we set the basic reproduction number *R*_0_, defined as the number of secondary infections caused by a single infected individual in an otherwise fully susceptible population [61], to 2. In all figures, we show median values over 500 stochastic simulation runs, along with their interquartile ranges (IQRs).

### Scenario 1: Concurrent vaccinations and epidemics

**Fig 1:**
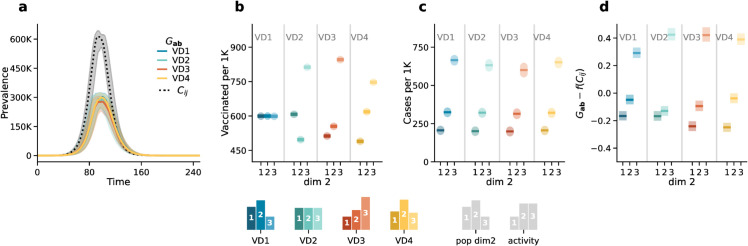
Scenario 1: epidemic outcomes. Panel *a* displays the number of infected individuals over time. The dotted black line corresponds to the outcome of the age-stratified model (i.e., *C*_*ij*_), and the solid colored lines correspond to the outcomes of the generalized model (i.e., $G_{ab}$) in the three different vaccination distributions (i.e., *VD*1,*VD*2,*VD*3, *VD*4). Panel *b* shows the percentage of vaccinated individuals in the three subgroups (i.e., *dim2*). Panel *c* shows the attack rate by the second dimension predicted by the generalized model. Panel *d* shows the difference between the attack rates predicted by the generalized model and those estimated from the aggregate output of the age-stratified model *f*(*C*_*ij*_) by the second dimension. Results refer to the median of 500 runs with IQRs (shaded areas). Epidemiological parameters: transmissibility Γ=0.25, recovery rate Ψ=0.4, vaccine efficacy against infection *g*_1_ = 0.6, basic reproductive number *R*_0_ = 2. Simulations start with *I*_0_ = 100 initial infectious seeds.

In Fig 1*A*, we begin by observing the epidemic wave under different vaccination distributions (i.e., *VD*1, *VD*2, *VD*3, *VD*4) as predicted by the generalized model (colored solid lines) and an age-stratified model (black dashed line). The first observation is that, as expected from Ref [52], the generalized model’s projections significantly differ from those of the age-stratified model. Indeed, the latter overestimates the total number of infected individuals. The discrepancy is due to the distribution of activity, assortativity and population among subgroups. Second, the total number of infections projected by the generalized model varies slightly across the different vaccination uptake distributions, although no substantial differences are visually apparent from the epidemic curves. In Fig 1*B*, we show the percentage of vaccinated individuals across the three population subgroups stratified by the second dimension (i.e., *dim2*). The panel illustrates how the different vaccination distributions perform. Under the random distribution (i.e., *VD*1), the three subgroups receive vaccines equally. Under the even distribution (i.e., *VD*2), group 3 has the highest vaccination coverage, followed by group 1, and the lowest in group 2. In both the activity based distribution (i.e., *VD*3) and the realistic distribution (i.e., *VD*4), vaccination coverage is highest in group 3, followed by group 2, and lowest in group 1.

The generalized model allows us to estimate the attack rate across the three socioeconomic subgroups by summing over all age groups. For example, to compute the total number of infected individuals in subgroup *α* at time *t*, we can sum over all age groups i.e., $I_{\alpha }(t)=\sum_{i}I_{i,\alpha }(t)$. Exploiting this feature of the generalized model, in panel *C* of Fig 1, we examine the attack rates predicted by the generalized model by subgroup for each vaccination distribution. Interestingly, the third group consistently appears as the most affected, while the first group remains the least infected across all vaccination distributions. This is solely due to the differences in activity levels: as the third group is the most active, it is always the most infected relative to its population size.

Finally, we investigate whether, and to what extent, it is possible to predict these outcomes from models that do not explicitly account for SES differences. First, we point out that infections across subgroups of the population, not explicitly considered by a model, can be estimated only *a posteriori* by leveraging outputs of the age-stratified model, such as attack rates for vaccinated ($AR_{V}$) and non-vaccinated ($AR_{NV}$) individuals. We developed a technique to estimate the number of infections within each subgroup *α* by multiplying the attack rates by the total number of vaccinated and non-vaccinated individuals in each subgroup at time *t*. For further details on this method, we refer the reader to the Methods Section and S1 Text Sect 6.1. This technique has two significant limitations: 1) it assumes precise knowledge of the number of vaccinated individuals in each subgroup defined by age and SES, which might not be realistic in practice, 2) it cannot, by definition, capture potentially different dynamics such as different peak times across subgroups unseen by the model.

In panel *d* of Fig 1 we show the difference between the number of cases in each subgroup projected by the generalized model ${𝐆}_{ab}$ and estimated from an age-stratified model $f({𝐂}_{ij})$. We note how *f* describes the *a posteriori* estimation of the attack rate from traditional models featuring only age-structured contact matrices *C*_*ij*_. The differences are consistently away from zero. Indeed, the attack rate within each subgroup results from the interplay among activity levels, contact patterns, population distribution, and vaccination coverage. This cannot be captured by traditional models that do not explicitly account for these factors. In Sect 7 of S1 Text, we further explore these results by running the same analysis under different vaccination distribution settings and a homogeneous mixing case. Overall, we observe the same qualitative findings.

#### The interplay between vaccinations and NPIs.

We now investigate how the interplay between heterogeneities in adherence to NPIs and vaccination distribution among subgroups affects epidemic outcomes. We explore hypothetical cases where a population modifies its behavior in response to the introduction of NPIs during an ongoing vaccination campaign. We assume that, to reduce contacts, NPIs are introduced 65 days after the onset of the epidemic, leading to a 20% overall reduction in contact rates, though with variations across subgroups. Indeed, we assume that the ability to adhere to the NPIs is associated with membership in a particular population group. Specifically, we explore a case where people in the first group (i.e., experiencing the lowest SES) cannot afford to protect themselves as the other two groups. We assume that the NPIs introduce changes in both assortativity and activity. Specifically, due to the NPIs, 50%, 60%, and 70% of the contacts for the first, second, and third groups, respectively, take place within their group. Thus, assortativity increases across all groups, with the second and third groups experiencing the most significant rise. Additionally, NPIs shift activity levels to 40%, 25%, and 35% for the first, second, and third groups, respectively. This results in a decrease in activity for the second and third groups, while the first group experiences a relative increase, further exacerbating its vulnerability. Following the same logic as before, we study the effects of four different vaccination uptake distributions. To isolate the impact of heterogenities across SES we neglect the general dependence between NPIs adoption and age [30].

**Fig 2:**
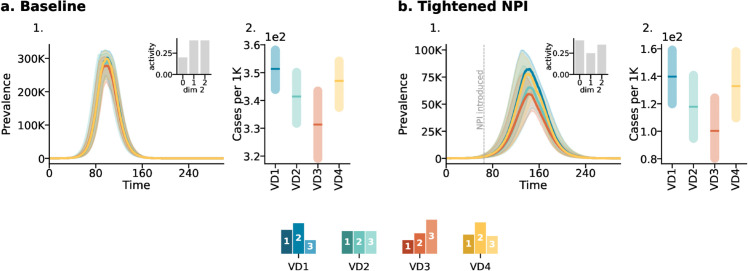
Scenario 1: The impact of NPIs under different vaccination distributions. Panel *a* refers to the baseline case. The inset depicts the activity distribution along the second dimension. The first column shows the prevalence, while the second column shows the attack rates per 1000. Panel *b* follows the same structure but refers to the case where NPIs are introduced, reducing contacts by 20%. The epidemic starts at *t*_*epi*_ = 0 with the onset of vaccination. NPIs are introduced 65 days after the epidemic onset. Results refer to the median of 500 runs with IQRs (shaded areas). Epidemiological parameters: transmissibility Γ=0.25, recovery rate Ψ=0.4, vaccine efficacy against infection *g*_1_ = 0.6, and basic reproductive number *R*_0_ = 2. Simulations begin with *I*_0_ = 100 initial infected seeds.

In Fig 2*A*, we report the results in a baseline case where the disease spreads unmitigated by any NPIs. The first column of panel Fig 2*A* displays the prevalence over time, while the second column shows the corresponding attack rates per 1000. While the differences between attack rates under different vaccination distributions are small, from panel 2, is visible that activity based distribution (i.e., *VD*3) is the most effective, leading to a higher reduction of cases. In contrast, the even and realistic distribution (*VD*2 and *VD*4) are less effective, with the random distribution (*VD*1) being the least effective.

As shown in Fig 2*B*, when NPIs are introduced, the attack rate of the different SES changes (see S1 Text Sect 8 for more information) thus influencing the relative effectiveness of vaccinations. In this scenario, despite variations in how vaccines are allocated among subgroups, the ranking of vaccination distribution effectiveness remains consistent. However, differences in epidemic curves are now observed. Specifically, *VD*3 and *VD*2 distributions lead to substantially lower peaks compared to *VD*1 and *VD*4 relative to the baseline scenario. For completeness, in S1 Text (see Sect 8), we investigate the effect of mortality by modeling the daily number of deaths. The results show the same trend.

### Scenario 2: Vaccinations completed before epidemics

In this scenario, we explore the epidemic dynamics assuming the vaccination rollout is already completed before the onset of the epidemic. This represents situations akin to a post-vaccination wave of infection, where the majority of the population has received vaccines, but a virus continues to circulate, possibly due to new variants and/or waning of natural immunity. We note how we do not consider waning in vaccine’s efficacy over time.

**Fig 3:**
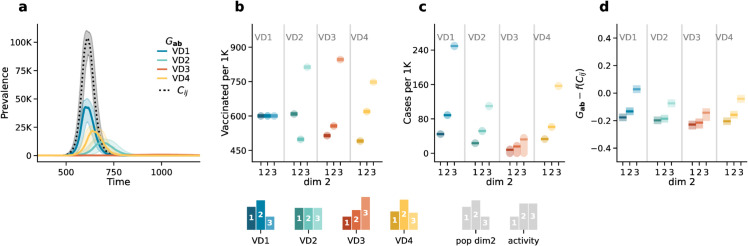
Scenario 2. Epidemic outcomes: Panel *a* displays the prevalence over time. The dotted black line corresponds to the outcome of the age-stratified model (i.e., ${𝐂}_{ij}$), and the solid colored lines correspond to the outcomes of the generalized model (i.e., ${𝐆}_{ab}$) in the three different vaccination distributions. Panel *b* shows the percentage of vaccinated individuals in the three subgroups (i.e., *dim2*). Panel *c* shows the attack rates per 1000 by the second dimension projected by the generalized model. Panel *d* shows the difference between the attack rates projected by the generalized model and those estimated from the aggregate output of the age-stratified model $f({𝐂}_{ij})$ by the second dimension. Results refer to the median of 500 runs with IQRs (shaded areas). Epidemiological parameters: transmissibility Γ=0.25, recovery rate Ψ=0.4, vaccine efficacy against infection *g*_1_ = 0.6, basic reproductive number *R*_0_ = 2. Simulations start with *I*_0_ = 100 initial infectious seeds.

Following the structure of the previous scenario, Fig 3*A* illustrates the number of infected individuals over time for the four vaccination distributions, as projected by the generalized model (see colored solid lines) and the traditional age-stratified model (see black dotted line). Also in this scenario, the generalized model projects different epidemic outcomes compared to the age-stratified model. Additionally, the differences between epidemic outcomes across vaccination distributions are more pronounced. This is because the epidemic starts only after the vaccinations have been fully implemented, maximizing the impact of the different vaccination distributions.

Notably, the activity based distribution (i.e., *VD*3) is the most effective vaccination strategy, resulting in a significantly lower number of infections. Interestingly, the second most effective strategy is the even distribution (i.e., *VD*2), followed by the realistic distribution (i.e., *VD*4). This result is explained by the higher vaccine coverage achieved in group 3 under these schemes. Indeed, the effectiveness of the vaccination strategies appears to be primarily driven by the level of protection of group 3, which is the most socially active subgroup. In contrast, the random distribution (i.e., *VD*1) provides insufficient coverage to this group—despite it being both the most active and the smallest—resulting in the least effective vaccination outcome and the highest overall prevalence.

Fig 3*C* breaks down the attack rates by SES, showing that even in this scenario, the most active group (i.e., group 3) experiences the highest number of infections relative to their population size, particularly under the random vaccine distribution. This confirms that, despite overall high vaccination coverage, specific subgroups can still disproportionately contribute to the spread of the virus.

In Fig 3*D*, we compare the predictions of the generalized model (i.e., ${𝐆}_{ab}$) with those derived from the age-stratified model (i.e., ${𝐂}_{ij}$) using the same technique discussed above (see Materials and methods and S1 Text Sect 6.1). Once again, we observe significant discrepancies between the two methods. Hence, even when the vaccination is completed before the start of a new wave of infection, explicitly accounting for the stratification of contacts across SES is important.

Overall, these results highlight the importance of explicitly incorporating subgroup-specific dimensions, such as socioeconomic status, into epidemic models.

In Sect 7 of S1 Text, we further explore these results by conducting a robustness analysis considering different vaccination distribution settings. In particular, we consider a case in which age is ignored in vaccination distribution and another that assumes homogeneous mixing between sub-groups. Notably, in the latter, we find that the difference between the projected attack rate from aggregate model output (i.e., $f({𝐂}_{ij})$) and those gathered from the generalized model (i.e., ${𝐆}_{ab}$) is consistently 0. This implies that in case of homogeneous mixing and when the overlap between vaccinations and epidemics is zero (i.e., vaccinations are completed before the start of the epidemic wave), the differences across sub-groups can also be obtained *a posteriori* from models that neglected this stratification.

#### The interplay between vaccinations and NPIs.

In this scenario, the population is already fully vaccinated before the start of the epidemic. We assume that before the start of the vaccination, some NPIs were in place, and we consider a situation where NPIs are then relaxed, leading to a 20% overall increase in contact rates. As before, we assume that the ability to adhere to NPIs varies across subgroups, with the variations remaining consistent with those depicted in Fig 2. The NPIs are reduced 65 days after the onset of the epidemic.

**Fig 4:**
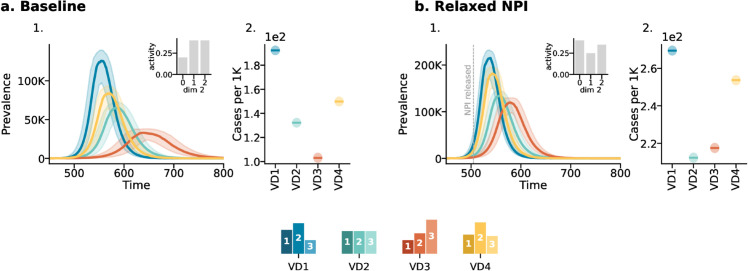
Scenario 2: The impact of NPIs under different vaccination distributions. Panel *a* refers to the baseline case, with insets depicting the activity distribution along the second dimension. The first column shows the prevalence as function of time, while the second column shows the attack rate scaled by 1000. Panel *b* follows the same structure but refers to the case where NPIs are released, increasing contacts by 20%. NPIs are adjusted 65 days after the epidemic onset. Results refer to the median of 500 runs with IQRs (shaded areas). Epidemiological parameters: transmissibility Γ=0.25, recovery rate Ψ=0.4, vaccine efficacy against infection *g*_1_ = 0.6, and basic reproductive number *R*_0_ = 2. Simulations begin with *I*_0_ = 100 initial infected seeds.

In the baseline case (see Fig 4*A*), although the different impact of vaccination distributions is more pronounced compared to the previous case, the relative effectiveness of the vaccination distributions follows the same order of the previous scenario. Indeed, the random distribution (*VD*1) is the least effective while the activity based (*VD*3) distribution is the most effective. The even (*VD*2) and realistic (*VD*4) distributions are in between these two, with the latter less effective than the former.

When NPIs are relaxed (see Fig 4*B*), the attack rate across SES groups changes according to the new activity distribution (See S1 Text Sect 8), which in turn affects the effectiveness of the vaccination strategies. In this scenario, the relative ranking of the strategies remains unchanged. However, the differences between strategies become more nuanced. Notably, while the activity based distribution is able to nearly suppress the epidemic under baseline conditions, its impact is now less pronounced and more comparable to that of the even distribution. These simulations highlight how the interplay between NPIs and vaccination distributions influences epidemic outcomes, with effectiveness depending on the timing and intensity of interventions. Also in this case, in S1 Text, we investigate the effect of mortality (see S1 Text Sect 8 for the corresponding results). Interestingly, when NPIs are relaxed, the activity-based distribution slightly surpasses the even distribution in terms of mortality rate.

### Hungarian contact data

We applied the model to empirical data describing social contacts stratified by age and one SES variable (i.e., self-perceived wealth with respect to the average) in Hungary during the COVID-19 Pandemic. The data has been collected via computer-assisted surveys from 1000 respondents describing a representative sample of the Hungarian adult population in terms of gender, age, education level, and type of settlement [53,54]. To build these matrices we used two contact diaries collected in April and November 2021, respectively, just before the third and the fourth wave of the COVID-19 Pandemic in Hungary. We refer the reader to the Materials and methods Section and Sects 1.2 and 9 of S1 Text for more details about the data and the Hungarian contact matrices.

**Fig 5:**
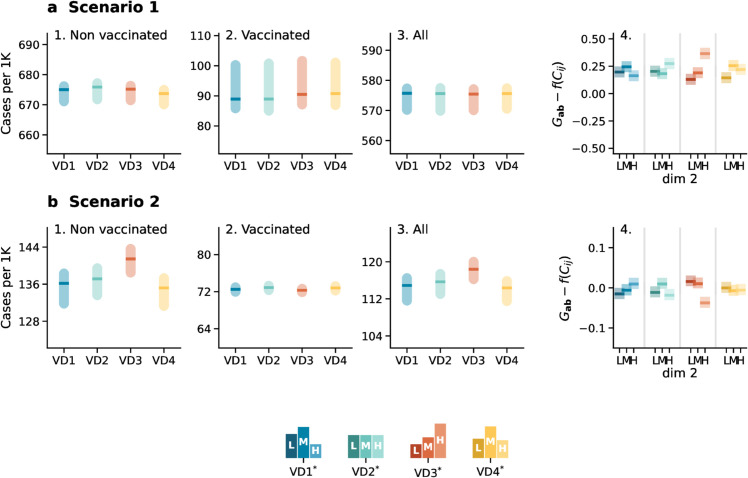
Epidemic outcomes under different vaccination distributions and real contact data. Panels in *a* 1–3 refer to Scenario 1 and display the number of cases per 1000 stratified by vaccination status (1. Non vaccinated, 2. Vaccinated, and 3. All) and under different vaccination distributions (i.e., *VD*1*,*VD*2*,*VD*3*,*VD*4*). Panel a 4 shows the difference between the attack rates predicted by the generalized model ${𝐆}_{ab}$ and those estimated from the aggregate output of the age-stratified model $f({𝐂}_{ij})$ by SES. Panel *b* shows the corresponding numbers for Scenario 2. Results refer to the median of 500 runs with IQRs (shaded areas). Epidemiological parameters: transmissibility Γ=0.25, recovery rate Ψ=0.4, vaccine efficacy against infection *g*_1_ = 0.6, and basic reproductive number *R*_0_ = 2.7 and *R*_0_ = 2, respectively for Scenario 1 and Scenario 2. Simulations start with *I*_0_ = 100 initial infectious seeds.

In Fig 5, we present the results for the Hungarian case, respectively for Scenario 1 (panel *A*) and Scenario 2 (panel *B*). In each panel, in subplots 1–3, we show the number of cases per 1000 individuals stratified by the non-vaccinated (1. *Non-vaccinated*) and vaccinated (2. *Vaccinated*) sub-populations and overall (3. All). To facilitate straightforward visual comparison between outcomes, the *y-axis* of the three subplots in each panel is set to span the same range. In other words, the difference between the maximum and minimum values on the *y-axis* is fixed across all subplots within the same panel.

In Scenario 1, the population was initialized with 20% of individuals being immune, representing those who had residual immunity. Vaccination was assumed to have started simultaneously with the onset of the epidemic, setting the day of the epidemic as time $t_{\text{epi}}=0$. For Scenario 2, the population was initialized with 40% of individuals being immune, reflecting a higher level of residual immunity. In this case, the epidemic was set to begin at $t_{\text{epi}}=220$ when ≈55% of the population is vaccinated, which implies 220 days had passed since the start of the vaccination campaign before the epidemic started. This adjustment mirrors the same time gap observed between the start of the COVID-19 vaccination campaign and the onset of the fourth epidemic wave in Hungary.

We apply the same vaccination strategies as in the synthetic scenarios. However, in the real-world data, individuals of different SES levels tend to have different age distributions (e.g., lower SES groups may have younger populations), but the strategies consider the same age-based prioritization across SES groups. As a result, the outcomes of these vaccination strategies do not preserve the same distribution across SES groups of the synthetic case (see S1 Text Sect 9.2). To highlight this, we marked the *VD* symbol with an asterisk.

The scenarios, modeled under different vaccination distributions (i.e., *VD*1*, *VD*2*, *VD*3* and *VD*4*), reveal distinct outcomes. In Scenario 1, the differences across vaccination distributions are small, as shown in Fig 5*A*1 − 3. This result can be explained by the fact that vaccination overlapped with a fairly fast epidemic. As a result, the effects of varying vaccination uptake are less visible, leading to similar outcomes.

In contrast, Scenario 2 (see Fig 5*B*) shows more pronounced differences among the vaccination distributions. Here, *VD*4* appears to be the most effective in reducing the attack rate. Interestingly, as shown in Sect 9.3 S1 Text, we find significant difference also in the mortality of the non-vaccinated population, indicating that even non-vaccinated individuals benefit indirectly from effective vaccination distributions.

In both panels, subplot 4 reports the difference in attack rates by SES obtained using the generalized model compared to a model that neglects SES. This difference was computed following the same methodology adopted in Figs 1 and 3. Notably, the divergence between the two models remains evident across both scenarios. However, in contrast to the results observed for the synthetic cases, the relative difference in Scenario 1 is now consistently positive across all SES groups, while in Scenario 2 it exhibits a mixed pattern, encompassing both positive and negative values.

## Discussion

The COVID-19 Pandemic was a stark reminder that inequalities in vaccine accessibility and in non-pharmaceutical interventions compliance can shape the trajectory of an epidemic. Building on the mathematical framework of generalized contact matrices, recently presented in Ref [52], here we studied the impact of these inequalities. We studied both synthetic and real generalized contact matrices investigating the interplay between the features of contact patterns and vaccination uptake across two dimensions: age and socioeconomic status (i.e., SES).

The timing of vaccinations relative to the epidemics is also a key factor [10]. Hence, we explored two scenarios. In the first, the epidemics and vaccinations begin simultaneously. In the second, the epidemics start in an almost fully vaccinated population. We considered a realistic daily allocation of vaccines to age groups taken from the Hungarian COVID-19 vaccination campaign in both scenarios. We analyze the impact on disease burden of four different vaccination distributions across SES groups. In the first, which acted as a baseline, vaccinations are set proportional to the size of each subgroup. In the second, subgroups are vaccinated equally, receiving the same share of vaccines. In the third, vaccinations are set considering the activity (i.e., share of overall contacts) of each subgroup. In the fourth, we considered realistic vaccination rates where subgroups’ uptakes are taken from real data (i.e., Hungarian COVID-19 vaccination).

Our results highlight the importance of using generalized models, that explicitly account for socioeconomic inequalities to accurately estimate the attack rate within different subgroups of the population. Indeed, we compared the attack rates predicted by generalized models in each subgroup of the population (e.g., SES) with those inferred *a posteriori* from the outputs of traditional age-stratified models. Interestingly, we found consistent differences between the two. Hence, disparities in vaccination uptake and contact patterns among subgroups make it challenging to estimate outcomes in subgroups of the population from models that do not consider these additional stratifications. These findings hold in both synthetic and real generalized contact matrices and underscore the importance of incorporating socioeconomic factors into epidemic models that aim to estimate the impact of vaccination campaigns.

Interestingly, in the case of synthetic generalized contact matrices, the third vaccination distribution strategy, which targets the most active subgroups of the population along the second dimension, is consistently the most effective in reducing the burden of the disease. The even distribution of vaccines is the second most effective, closely followed by the realistic distribution. The baseline, instead, leads to the worst outcomes. In the case of real generalized contact matrices, we found the differences among distribution strategies less apparent, but the realistic distribution emerges as the most effective strategy. The discrepancy between the results obtained considering different contact matrices underscores the interplay between the details of contact patterns (e.g., activity and assortativity) and vaccination uptake. As these features are, in general, country dependent, our results suggest caution when extrapolating analysis across different contexts. Furthermore, as both synthetic and real generalized contact matrices have the exact stratification across the age dimension, these results highlight, one more time, the importance of models able to explicitly capture additional dimensions of the population under study.

In both scenarios, we studied how heterogeneities in adherence to NPIs and vaccination distribution among subgroups affect epidemic outcomes. In particular, in the first scenario, we imagined that NPIs are introduced a given number of days after the start of an epidemic wave. In the second, instead, we imagined that NPIs are relaxed a given number of days after the start of the new wave of infections. We introduced heterogeneities in adherence to NPIs and in their relaxation, assuming that the ability to implement or relax them is a function of membership to a particular sub-group. In particular, we assumed that, due to NPIs, assortativity would increase (especially in mid and high SES) and that the activity levels would shift in such a way that the people experiencing low SES would see a relative increase in the share of total contacts with respect to mid and high SES. Consistently, in the second case we imagined changes in assortativity and activity as a function of SES but here variations are linked to an increase in contacts rather than to a reduction. Interestingly, the vaccination distribution that targets activity is found to be the most effective. Furthermore, we found that, while the prevalence profiles are affected by the vaccine distribution used (especially in the second scenario), the relative effectiveness of strategies remains unchanged by the implementation or relaxation of NPIs.

As detailed in S1 Text, the results are affected by the choice of *R*_0_ and the timing of the epidemics’ onset ($t_{\text{epi}}$). Indeed, adjusting these two parameters modulates the overlap between epidemic waves and vaccinations, and allows to explore situations in between the two scenarios reported here. The analysis showed that, as we fix *R*_0_ and increase $t_{\text{epi}}$—thus reducing the overlap between epidemic waves and vaccinations—the difference between the two methods converges to a stable value, typically different from zero. A similar trend occurs when we increase *R*_0_ with $t_{\text{epi}}$ set to zero. For further details, see S1 Text 7.3. Interestingly, these results are in line with analysis conducted by Larsen et al. in Ref [10] where, in the context of COVID-19, the role of the timing and relative speed of vaccination has been found critical to reduce the disease burden across all subgroups, more than final coverage and differences of uptake across SES.

It is important to acknowledge the limitations of our work. First, we note that our study relies solely on what-if scenarios designed to mimic real-world cases. We did not calibrate the model to epidemic data. The model used to generate synthetic generalized contact matrices, developed in Ref [52], was not designed to replicate empirical data but to provide a flexible framework for exploration. The model neglects correlations between SES and age in assortativity and activity levels, as well as the modulating role of other variables. The modeling of NPIs was guided by simplicity rather than realism. Indeed, our aim was to showcase the potential interactions between vaccination uptake, contact patterns, and NPIs, and to demonstrate their effects on the epidemic outcome, while providing a framework that can be adapted to different settings. We neglected the dependence between NPIs adherence and age to focus solely on the role of SES. Though the model starts from real age pyramids and real age-structured contact matrices, in each age group it is characterised by the same split across SES. Finally, a simplification was made in the modeling of the vaccination protocol by considering only a single dose that becomes immediately effective. More work is needed to address these limitations and extend the scope of the research presented here. To this end, modeling efforts should be assisted by progress in data collection and sharing. Indeed, most of the relevant data is now disaggregated only by age, thus limiting the scope and application of generalized models.

Overall, our study shows that incorporating socioeconomic heterogeneities into epidemic models allows for more accurate estimations of the attack rate across different population subgroups. By accounting for inequalities in both contact patterns and vaccination uptake, our model provides a nuanced view of how these factors interact to influence epidemic dynamics. Our framework offers a straightforward, easy-to-implement, approach that enables estimating attack rate across different subgroups. This method could assist public health officials in tailoring vaccination campaigns and other interventions more effectively, ultimately reducing the unequal impact of epidemics on vulnerable populations.

## Materials and methods

### Ethics statement

The data used to build the real generalized contact matrices used are obtained from the MASZK survey study [53,54]. The data collection adhered to European and Hungarian privacy regulations, approved by the Hungarian National Authority for Data Protection and Freedom of Information [62], as well as the Health Science Council Scientific and Research Ethics Committee (resolution number IV/3073-1/2021/EKU).

### Epidemiological model

We consider a Susceptible-Exposed-Infectious-Recovered (SEIR) compartmental model with vaccination where susceptible (*S*) are healthy individuals at risk of infection, exposed (*E*) are infected but not yet infectious, infectious (*I*) can spread the disease and recovered (*R*) are no longer infectious nor susceptible to the disease [63]. All compartments are then further stratified between non-vaccinated (NV) and vaccinated (V) individuals. In S1 Text, we also model mortality by adding a death (*D*) compartment to the model.

We focus on two models that primarily differ in how they represent individual interactions: the age-stratified and the generalized model (see Sect 4 of S1 Text for further details).

#### Age-stratified model.

The age-stratified model uses contact matrices stratified by age **C** to account for the differences in contact patterns among various age groups. The element ${𝐂}_{ij}$ quantifies the average number of contacts that an individual in age bracket *i* has with individuals in age group *j* within a certain time window [64–66]. The population is divided into age brackets so that $N=\sum_{i=1}^{K}N_{i}$. The variables *N*_*i*_ capture the number of individuals in age group *i* while *K* indicates the number of different age groups.

#### Generalized model.

The generalized model extends the concept of age-stratified contact matrices to include multiple dimensions, represented by generalized contact matrices **G**. In more detail, we describe the generalized contact matrices as ${𝐆}_{𝐚,𝐛}$, where 𝐚=(i,α) and 𝐛=(j,β) are tuples (i.e., index vectors) representing individuals’ membership to each category. With these matrices we can, for example, capture contact stratification according to age and income. In this case, ${𝐆}_{𝐚,𝐛}$ would then describe the average number of contacts that an individual in age bracket *i* and income *α* has with people in age group *j* and income *β*, in a given time window. We refer the reader to S1 Text Sect 2 for more details.

#### Vaccinations.

Vaccination data on the daily doses administered is available, at the lower level, by age groups (see S1 Text Sect 1.1), which we distribute among subgroups (SES) according to a given distribution $P_{vax}(\alpha )$, which indicates the proportion of vaccines allocated to each subgroup. Let $\Omega_{i}(t)$ represent the daily administered doses at time *t* for age group *i*. The number of vaccines administered to a subgroup *α* is then $\Omega_{i,\alpha }(t)=\Omega_{i}(t)$ ⋅ $P_{vax}(\alpha )$. Each subgroup can be vaccinated up to 95%. If this limit is reached, any excess vaccinations are redistributed randomly within that particular age group.

#### Numerical simulations.

We developed a stochastic, discrete-time, compartmental model where the transitions among compartments are simulated through chain binomial processes. In particular, at time step *t* the number of individuals in group **a** and compartment *X* transiting to compartment *Y* is sampled from $PrBin(X_{𝐚}(t),p_{X_{𝐚}\to Y_{𝐚}}(t))$. Where, PrBin(n,p) denotes a probabilistic sampling from a binomial distribution with *n* trials and success probability *p*, which represents the number of individuals making the transition from compartment *X* to compartment *Y* in group **a** at time *t*, and $p_{X_{𝐚}\to Y_{𝐚}}(t)$ is the transition probability (see S1 Text Sect 5 for further details).

### Estimating the attack rate of subgroups from age-stratified models

The attack rate (AR) is defined as the fraction of all individuals in a susceptible population who have been infected before a specific time *t* during an epidemic outbreak. In a compartmental SEIR model, the attack rate at time *t* can be computed as follows:

$$AR(t)=\frac{M_{S\to E}(t)}{N}$$ (1)

where $M_{\left(S\to E\right)}$ indicates the number of transitions from the susceptible (*S*) to the exposed (*E*) state up to time *t*, and *N* represents the total number of individuals in the population (see Sect 6 of S1 Text for further details).

The attack rate for specific subgroups *α*, defined along the second dimension, ($AR_{\alpha }$) can be estimated using outputs from a model stratified solely by age. We refer to the output of such estimation as $f({𝐂}_{ij})$. As a first step we need to infer the attack rate within each age group in the subgroup (i,α) and subsequently aggregate these age-specific results to obtain $AR_{\alpha }$. This requires estimating the number of susceptible individuals in each subgroup who became exposed during the epidemic i.e., $M_{S_{i,\alpha }\to E_{i,\alpha }}$. This involves calculating the attack rates for vaccinated $AR_{Vi}$ and non-vaccinated $AR_{NVi}$ individuals, in each age group *i*, from the age-stratified model (see S1 Text Sect 6.1 for further details on this calculation). Therefore, we can multiply $AR_{NVi}$ and $AR_{Vi}$ by the total number of non-vaccinated and vaccinated individuals in each group (i,α) up to time *t*, taking into account the transitions between non-vaccinated and vaccinated states over time. The equation used for this calculation reads as follows:

$$M_{S_{i,\alpha }\to E_{i,\alpha }}(t)=AR_{Vi}(t)M_{S_{NVi,\alpha }\to S_{Vi,\alpha }}(t)+AR_{NVi}(t)\left(N_{i,\alpha }-M_{S_{NVi,\alpha }\to S_{Vi,\alpha }}(t)\right),$$ (2)

where $AR_{Vi}(t)$ and $AR_{NVi}(t)$ represent respectively the attack rates for vaccinated and non-vaccinated individuals in age group *i* and $M_{S_{NVi,\alpha }\to S_{Vi,\alpha }}(t)$ denotes the number of susceptible individuals in groups (i,α) who were vaccinated up to time *t*. The attack rate for subgroups (i,α) can be then calculated by dividing Eq (2) for the population size as follows:

$$AR_{i,\alpha }(t)=\frac{AR_{Vi}(t)M_{S_{NVi,\alpha }\to S_{Vi,\alpha }}(t)+AR_{NVi}(t)\left(N_{i,\alpha }-M_{S_{NVi,\alpha }\to S_{Vi,\alpha }}(t)\right)}{N_{i,\alpha }}$$ (3)

We can then aggregate by age groups Eq (3) and divide by the total number of individuals in subgroup *α* as follows:

$$AR_{\alpha }(t)=\frac{\sum_{i}AR_{Vi}(t)M_{S_{NVi\alpha }\to S_{Vi\alpha }}(t)+AR_{NVi}(t)\left(N_{i,\alpha }-M_{S_{NVi\alpha }\to S_{Vi\alpha }}(t)\right)}{N_{\alpha }}$$ (4)

See S1 Text Sect 6.1 for further details.

### Real generalized contact matrices

The real generalized contact matrices used are obtained from the MASZK survey study [53,54], a large data collection effort on social mixing patterns made during the COVID-19 pandemic, conducted in Hungary from April 2020 to July 2022. The study recorded 26 monthly cross-sectional anonymous phone surveys using Computer Assisted Telephone Interview (CATI) methodology, with a nationally representative sample of at least 1000 participants each month. The recorded population was representative in terms of gender, age, education level, and type of settlement. Sampling errors were further corrected by post-stratification weights. See S1 Text Sects 1.2 and 9 for further details).

## Supporting information

S1 Text Supplementary analyses for the models and results. In this supplementary file (PDF), we present additional analyses and results of our work.
(PDF)
