# Supplementary material for: Social inequalities in vaccine coverage and their effects on epidemic spreading
Source: PLoS Comput Biol. 2025 Oct 13;21(10):e1013585. doi: 10.1371/journal.pcbi.1013585 (PMC12533974; doi:10.1371/journal.pcbi.1013585)
Supplement: S1 Text — In this supplementary file (PDF), we present additional analyses and results of our work. (PDF) [file pcbi.1013585.s001.pdf]

Supplementary Information

Social inequalities in vaccine coverage and their effects on epidemic spreading

Adriana Manna[1], Márton Karsai[1,2], Nicola Perra[3,4*]

[1] Department of Network and Data Science, Central European University, Vienna, Austria
[2] National Laboratory for Health Security, HUN-REN Rényi Institute of Mathematics, Budapest, Hungary
[3] School of Mathematical Sciences, Queen Mary University of London, London, UK
[4] The Alan Turing Institute, London, UK
*n.perra@qmul.ac.uk

October 4, 2025

Contents

1 Data **3**
1.1 Vaccination data . . . 3
1.2 Contact data . . . 3

2 Generalized contact matrices **4**
2.1 Synthetic generalized contact matrices . . . 5
2.2 Random mixing regime . . . 6

3 Parameters used for Figures 1–4 **7**

4 Epidemic models **7**
4.1 Age-stratification . . . 8
4.1.1 Derivation of R_0 . . . 8
4.2 Generalized models . . . 9
4.2.1 Derivation of R_0 . . . 9
4.2.2 Modeling vaccination uptake among the second dimension . . . 9

5 Numerical simulations **9**
5.1 Pseudo-Code . . . 10

6 Computing attack rates **11**
6.1 Estimating the attack rate of subgroups from age-stratified models . . . 13

7 Robustness analysis for Figs 1 and 3 **14**
7.1 Assortative mixing regime . . . 14
7.1.1 Vaccination stratified only by *dim2* . . . 14
7.2 Random mixing regime . . . 16
7.2.1 Vaccination stratified by age and *dim2* . . . 16
7.2.2 Vaccination stratified only by *dim2* . . . 18
7.3 Sensitivity to varying t_{epi} and R_0 . . . 20
7.3.1 Vaccination stratified by age and *dim2* . . . 20
7.3.2 Vaccination stratified only by *dim2* . . . 20

8 Non pharmaceutical interventions **22**

9 Hungarian contact data **25**
9.1 Assortativity by SES 25
9.2 Vaccination distributions using real-world contact matrices 26
9.3 Epidemic outcomes using real-world contact matrices 26

1 Data

The age-stratified data on vaccination, contact patterns, and population used in this work are based on data collected in Hungary. As presented in the following sections, COVID-19 vaccination data come from the Our World in Data (OWID) database [1], while contact and population data are sourced from the MASZK study [2, 3].

1.1 Vaccination data

Data on the number of vaccines administered have been sourced from Our World in Data [1]. Figure A presents the number of COVID-19 vaccines administered daily by age in Hungary.

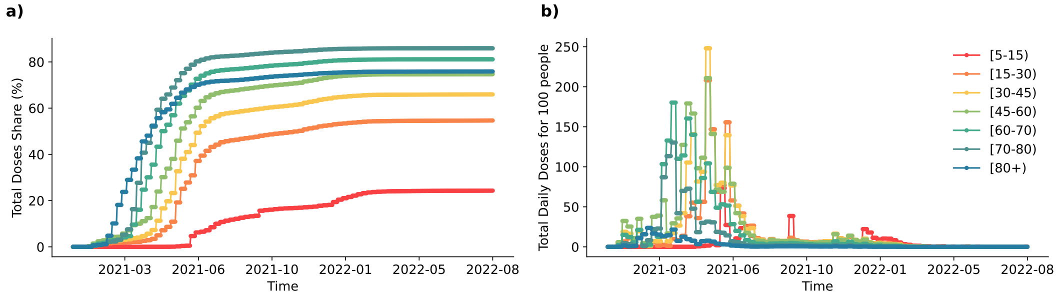

Figure A: **Hungary COVID-19 vaccination. a.** Share of people who completed the initial COVID-19 vaccination protocol by age **b.** Total Daily Dose for 100 people by age.

1.2 Contact data

In this study we used data coming from the MASZK survey study [2, 3], a large data collection effort on social mixing patterns made during the COVID-19 pandemic, conducted in Hungary from April 2020 to July 2022. The study involved 26 monthly cross-sectional anonymous phone surveys using Computer Assisted Telephone Interview (CATI) methodology, with a nationally representative sample of 1000 participants each month. The recorded population was representative in terms of gender, age, education level, and type of settlement. Sampling errors were further corrected by post-stratification weights. The data collection adhered to European and Hungarian privacy regulations, approved by the Hungarian National Authority for Data Protection and Freedom of Information [4], as well as the Health Science Council Scientific and Research Ethics Committee (resolution number IV/3073-1/2021/EKU).

Relevant to this study, the questionnaires recorded information about the *proxy social contacts*, defined as interactions where the respondent and a peer stayed within 2 meters for more than 15 minutes [5], at least one of them without wearing a mask. Approximate contact numbers were recorded between the respondents and their peers from different age groups of 0–4, 5–14, 15–29, 30–44, 45–59, 60–69, 70–79, and 80+. Contact data about underage children was collected by asking legal guardians to estimate daily contact patterns. Participants during the whole data collection were asked to report contacts referring to (*i*) the previous day and, during the first data collection campaigns (*ii*) an average pre-pandemic day (that we use for the analysis in Fig. 1-4 in the main text). Additionally, in three data collection waves: April 2021, November 2021, and June 2022, contacts have been collected in the form of diaries (data from April and November 2021 are used respectively for Scenario 1 and 2 of Fig. 5 of the main text). Participants were asked to list one by one the contacts they had on the previous day by providing some socio-demographic information about the contacts, such as their wealth situation. Beyond information on contacts before and during the pandemic, the MASZK dataset provided us with information on *social-demographic characteristics* of participants, such as their *perceived wealth situation*, *gender*, *vaccination status*, etc. To stratify the contact matrix by SES, we use the variable *perceived wealth situation*. Specifically, individuals were asked to report both their own perceived income and the perceived income of each person they had contact with, relative to the average, using a scale from 1 to 10. We classified *low-income* individuals as those who answered

from 1 to 4, *mid-income* as those who answered 5 or 6, and *high-income* as those who answered 7 or above. Although this variable reflects perceived income, for conciseness, we refer to it in the text as income. We acknowledge that there may be biases when participants report the perceived income of their contacts; however, this was the most feasible approach to collect such information at scale, as capturing socioeconomic dimensions within contact data represents a challenge in itself.

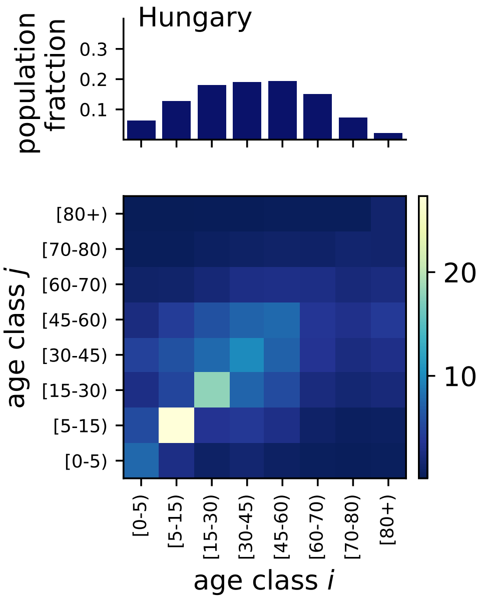

Figure B: **Hungarian population distribution and age contact matrix:** (1*st row*) Population distribution by age (2*nd row*) Age-contact matrix referred to the pre-pandemic period for Hungary.

2 Generalized contact matrices

Following Ref. [6] we define a generalized contact matrix as $\mathbf{G}_{\mathbf{a},\mathbf{b}}$, where $\mathbf{a} = (i, \alpha, \beta, \ldots, \gamma)$ and $\mathbf{b} = (j, \eta, \mu, \ldots, \xi)$ are tuples (i.e., index vectors) encoding the categorical membership of individuals across several dimensions. This representation allows to consider contacts stratified by age and other m dimensions, for example, income (α), and education level (β). In this case, $\mathbf{G}_{\mathbf{a},\mathbf{b}}$ would describe the average number of contacts that an individual in age bracket i, income α, and education β has with people in age group j, income η, and education μ in a given time frame.

The total number of contacts between individuals in age groups i and j is given by $\mathbf{R}_{ij} = \mathbf{C}_{ij}\mathbf{N}_i$. When using generalized contact matrices, this expression becomes $\mathbf{C}_{ij}\mathbf{N}_i = \sum_{\mathbf{a}'\mathbf{b}'} \mathbf{G}_{\mathbf{a}',\mathbf{b}'}\mathbf{N}_{\mathbf{a}'}$, where $\mathbf{a}' = \mathbf{a} - \{i\}$ and $\mathbf{b}' = \mathbf{b} - \{j\}$ are the index vectors capturing all dimensions except age. Since the matrices $\mathbf{G}$ has $m+1$ dimensions, contacts can be aggregated along any of these axes. For instance, we can compute the average number of contacts between individuals in SES group α and those in group β as:

$$\mathbf{C}_{\alpha\beta}\mathbf{N}_\alpha = \sum_{\mathbf{a}'\mathbf{b}'} \mathbf{G}_{\mathbf{a}',\mathbf{b}'}\mathbf{N}_{\mathbf{a}'} \quad (1)$$

where now $\mathbf{a}' = \mathbf{a} - \{\alpha\}$ and $\mathbf{b}' = \mathbf{b} - \{\beta\}$. Since the number and size of groups may differ depending on the chosen stratification, in general $\mathbf{C}_{ij} \neq \mathbf{C}_{\alpha\beta}$.

We denote with K the number of age groups, while with V_p the number of groups in each m other dimensions (i.e., $p \in [1, m]$). While the generalized matrix $\mathbf{G}$ can be naturally described as a multidimensional matrix, the use of $T = K \prod_{p=1}^{m} V_p$ index vectors pairs allows for flattened representation in a squared bi-dimensional matrix of size $T \times T$.

2.1 Synthetic generalized contact matrices

In Figures 1–4 of the main text we used synthetic generalized contact matrices featuring age and an additional dimension (e.g., SES). To create these matrices we followed the model proposed in Ref. [6].

The model starts from an empirical contact matrix $\mathbf{C}$ that encodes average contact rates between age groups i and j. The total number of contacts between individuals in those age groups can be written as $\mathbf{R}_{ij} = \mathbf{C}_{ij}\mathbf{N}_i$. This quantity is symmetric, since $\mathbf{R}_{ij} = \mathbf{R}_{ji} = \mathbf{C}_{ji}\mathbf{N}_j$. The model disaggregates $\mathbf{R}_{ij}$ into finer-grained values $(\mathbf{R_G})_{i\alpha,j\beta}$, representing the number of contacts between individuals of age i and SES α, and those of age j and SES β:

$$\mathbf{R}_{ij} = \sum_{\alpha,\beta}(\mathbf{R_G})_{i\alpha,j\beta}$$

To build these matrices, the model imposes two main conditions that ensure consistency with the empirical data and with the basic properties of contact processes:

- **Symmetry:** $(\mathbf{R_G})_{i\alpha,j\beta} = (\mathbf{R_G})_{j\beta,i\alpha}$.
  This condition ensures that the number of raw contacts between any two subgroups is the same in both directions. That is, the number of contacts from individuals of age group i and SES α to individuals of age group j and SES β must equal the number of contacts in the reverse direction. This mirrors the symmetry of the total contact matrix $\mathbf{R}$, where $\mathbf{R}_{ij} = \mathbf{R}_{ji}$, and is a necessary property in models where contacts are assumed to be reciprocal. This symmetry is imposed on the disaggregated structure of the contact matrix to reflect the mutual nature of interactions, even when stratifying by additional attributes such as SES.

- **Conservation of contacts:** $\sum_{\alpha,\beta}(\mathbf{R_G})_{i\alpha,j\beta} = \mathbf{R}_{ij}$.
  This condition guarantees that the generalized contact matrix is consistent with the empirical contact rates observed between age groups. The total number of contacts between age group i and age group j, when summed across all SES combinations, must equal the empirical value $\mathbf{R}_{ij} = \mathbf{C}_{ij}\mathbf{N}_i$. In other words, disaggregating contacts by SES should not alter the marginal totals provided by the age stratified matrix. This ensures compatibility with established contact data and allows the generalized model to preserve known contact patterns while introducing additional heterogeneity.

Assuming only one additional dimension, as we do in the main text, for any i and j the matrix $(\mathbf{R_G})_{i\alpha,j\beta}$ is of size $V_1 \times V_1$. In case, of $i \neq j$ the model needs to specify $W = V_1^2 - 1$ elements. For all $i = j$ instead, the symmetry of the matrix requires only $Y = V_1 + \frac{V_1(V_1-1)}{2} - 1$ elements. For all $V_1 \geq 2$ it is easy to show how $W > Y$, hence the matrix can be defined specifying W values. On top of the two constrains mentioned above, the model assumes that each SES (or more in general any additional dimension) is associated to a given 1) activity and 2) assortativity. In more details, the model assumes that for any given pair of i and j:

1. each SES α is responsible for P_α fraction of $\mathbf{R}_{ij}$ connections. The values of P_α set the activity of each SES and are defined such that $\sum_\alpha P_\alpha = 1$.

2. a fraction q_α of these are in-group connections (defined in the diagonal) and $1-q_\alpha$ are instead out-group links (taking place off-diagonal). These parameters controls the assortativity of connections within each group.

Case $i = j$. In this case, the matrices $(\mathbf{R_G})_{i\alpha,i\beta}$ can be conveniently written as

$$(\mathbf{R_G})_{i\alpha,i\beta} = R_{ii}\begin{pmatrix} q_1P_1 & p_{i1,i2} & p_{i1,i3} \\ p_{i1,i2} & q_2P_2 & p_{i2,i3} \\ p_{i1,i3} & p_{i2,i3} & q_3P_3 \end{pmatrix} \quad (2)$$

The values $p_{i\alpha,i\beta} \in [0,1]$ are defined such that $(\mathbf{R_G})_{i\alpha,i\beta} = \mathbf{R}_{ii}p_{i\alpha,i\beta}$. As the matrix needs to be symmetric, we wrote $p_{i1,i2}$ instead of $p_{i2,i1}$. The activity of each SES group P_α defines the fraction of contacts R_{ii} they are involved with. The assortativity q_α defines the fraction of these that are in-group (i.e., $\alpha = \beta$). Hence, the complementary fraction $(1 - q_\alpha)P_\alpha = \Pi_\alpha$ take place across different SES

groups. These constraints define a system of linear equations. For $V_1 = 3$, the system can be written as:

$$\begin{cases} p_{i1,i2} + p_{i1,i3} = \Pi_1 \\ p_{i1,i2} + p_{i2,i3} = \Pi_2 \\ p_{i1,i3} + p_{i2,i3} = \Pi_3 \end{cases} \quad \Rightarrow \quad \hat{\mathbf{p}} = \frac{1}{2}\begin{pmatrix} \Pi_1 + \Pi_2 - \Pi_3 \\ \Pi_1 + \Pi_3 - \Pi_2 \\ \Pi_2 + \Pi_3 - \Pi_1 \end{pmatrix}$$

If the resulting values are non-negative, the matrix $(\mathbf{R_G})_{i\alpha,i\beta}$ is fully specified. In other words, for $V_1 = 3$ the system is defined without the need of additional free parameters. If instead some of the values are negative, the parameters P_α and q_α must be adjusted to yield a physical (i.e., non-negative) solution.

As mentioned above, for any V_1, the number of free parameters is

$$Y = V_1 + \frac{V_1(V_1 - 1)}{2} - 1$$

For $V_1 = 3$ we have $Y = 5$. Hence, Y is equal to $2V_1 - 1$ which is the number of free parameters P_α and q_α defined by the activity and assortativity. In general, for $V_1 > 3$, additional $Y - (2V_1 - 1)$ parameters must be fixed to solve the underdetermined system.

Case $i < j$. When $i \neq j$, the block $(R_G)_{i\alpha,j\beta}$ is not symmetric under exchange of α and β, and must be defined separately. As written above, the number of parameters to set is:

$$W = V_1^2 - 1$$

In the case $V_1 = 3$, the following system applies:

$$\begin{cases} p_{i1,j2} + p_{i1,j3} = \Pi_1 \\ p_{i2,j1} + p_{i2,j3} = \Pi_2 \\ p_{i3,j1} + p_{i3,j2} = \Pi_3 \end{cases}$$

We can choose three entries (e.g., $p_{i1,j2}$, $p_{i2,j1}$, and $p_{i3,j1}$) as free parameters, and compute the others accordingly:

$$\begin{cases} p_{i1,j3} = \Pi_1 - p_{i1,j2} \\ p_{i2,j3} = \Pi_2 - p_{i2,j1} \\ p_{i3,j2} = \Pi_3 - p_{i3,j1} \end{cases}$$

To ensure non-negativity and interpretability, the free parameters can be defined as fractions:

$$p_{i\alpha,j\beta} = a_\alpha \Pi_\alpha \quad \text{with} \quad a_\alpha \in [0, 1]$$

The matrix $(\mathbf{R_G})_{j\beta,i\alpha}$ for $i > j$ is then obtained by transposing $(\mathbf{R_G})_{i\alpha,j\beta}$.

In summary, the model constructs generalized contact matrices that preserve the empirical age-stratification. The matrices are defined by interpretable parameters P_α (the activity) and q_α (the assortativity), plus, possibly, other free parameters according to the number of groups in the additional dimension. It is important to stress how the model sets the same parameters across all age pairs. Hence, it does not account for general relationships among age, activity, and assortativity.

2.2 Random mixing regime

By setting $P_\alpha = q_\alpha = N_\alpha/N$ (defining $N_\alpha = \sum_i N_{i\alpha}$) in each SES group α we obtain a *random mixing regime*. Indeed, in this case contacts between individuals are formed proportionally to group sizes, without any preference or assortative structure. As shown in Ref. [6] the contact structure is fully determined by the demographic distribution. In other words, the assumption of random mixing allows defining the generalized contact matrix without the need of additional parameters besides the population pyramid.

3 Parameters used for Figures 1–4

Here, we report the values of the activity, assortativity, and other free parameters used in the simulations (see Table A).

SES Group	**1**	**2**	**3**
Population distribution	35%	45%	20%
Assortativity	60%	50%	65%
Activity	20%	40%	40%
Free parameters	0.6	0.6	0.5

Table A: List of parameters used to generate Fig 2 in the main text.

In Fig C we show the generalized contact matrix with two dimensions: age and *dim2* that we used to generate Figs 1 and 4 of the main text.

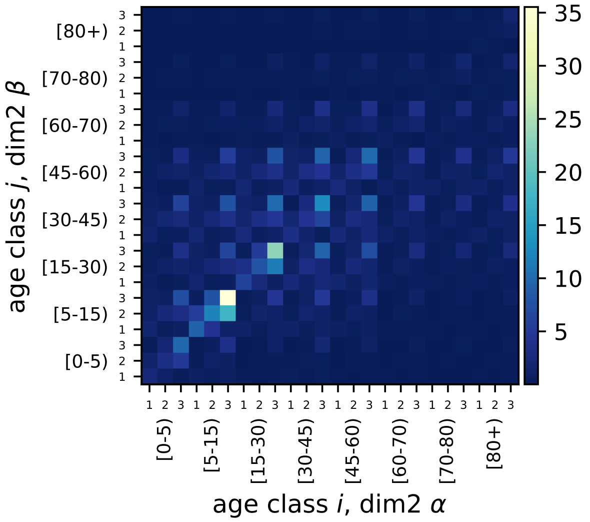

Figure C: **Generalized contact matrix with two dimensions: age and *dim2***

4 Epidemic models

This section provides a detailed description of the SEIR model with vaccination. Susceptible individuals, in contact with the infected, might be exposed to the virus with a rate driven by the force of infection Λ; exposed are not yet infectious and transition to the infected compartment with rate Ψ; infected individuals recover with rate Γ. Each compartment is divided between non-vaccinated NV and vaccinated V. Thus the total number of individuals in compartment X (i.e., $X = [S, E, I, R]$) is $X = X_{NV} + X_V$. Individuals who received one dose of vaccine move to the compartments denoted with the superscript V. We assume all individuals except for the infectious can receive the vaccine. The number of people that get vaccinated at each time step is defined by $\Omega(t)$, and it is distributed among S_{NV}, E_{NV}, R_{NV}, proportionally to their share in the population. The effectiveness of the vaccine is described by g_1 and g_2, which respectively represent efficacy against infection and death. Additionally, we assume that infected vaccinated individuals are less likely to further transmit the virus, this is modelled by the parameter VE in the force of infection. A detailed description of all epidemiological parameters is presented in Table B.

The formulation of this model varies depending on how the population structure and contact patterns are described. Below, we present two different models that we study.

Parameter	Description	Value
Λ_i	Force of infection	Computed as in Eq. (4) and (7)
ϕ	Transmissibility rate	Derived from Eq. (5) and (8)
Ψ	Rate of transition from exposed to infectious	$0.4\,\text{day}^{-1}$
Γ	Recovery rate	$0.25\,\text{day}^{-1}$
g_1	Vaccine efficacy against infection	0.6
g_2	Vaccine efficacy against death	0.8
VE	Reduction in transmission by vaccinated infected	0.4
$\Omega(t)$	Daily vaccination rate (time-dependent)	Computed from data

Table B: Epidemiological parameters used in the SEIR model with vaccination.

4.1 Age-stratification

Standard approaches to model the spreading of infectious diseases often acknowledge the stratification of contacts across age brackets. To this end, contact matrices $\mathbf{C}$ are introduced. The element $\mathbf{C}_{ij}$ quantifies the average number of contacts that an individual in age-bracket i has with individuals in age group j within a certain time window [7–9]. The population is divided into age brackets so that $N = \sum_{i=1}^{K} N_i$. The variables N_i capture the number of individuals in age group i while K indicates the number of different age groups. The SEIR model with vaccination that incorporates such matrices can be written as follows:

$$\begin{aligned}
d_t S_{NVi}(t) &= -\Lambda_i(t) S_{NVi}(t) - \Omega(t)_S, \\
d_t S_{Vi}(t) &= -(1-g_1)\Lambda_i(t) S_{Vi}(t) + \Omega(t)_S, \\
d_t E_{NVi}(t) &= \Lambda_i(t) S_{NVi}(t) - \Psi E_{NVi}(t) - \Omega(t)_E, \\
d_t E_{Vi}(t) &= (1-g_1)\Lambda_i(t) S_{Vi}(t) - \Psi E_{Vi}(t) + \Omega(t)_E, \\
d_t I_{NVi}(t) &= \Psi E_{NVi}(t) - \Gamma I_{NVi}(t), \\
d_t I_{Vi}(t) &= \Psi E_{Vi}(t) - \Gamma I_{Vi}(t), \\
d_t R_{NVi}(t) &= \Gamma I_{NVi}(t) - \Omega(t)_R, \\
d_t R_{Vi}(t) &= \Gamma I_{Vi}(t) + \Omega(t)_R,
\end{aligned} \tag{3}$$

where i is the index that describes the membership of individuals in the K age groups and the subscripts NV and V indicate respectively the non-vaccinated and vaccinated individual in each compartment. The force of infection is then defined as the per-capita rate at which susceptibles acquire infections:

$$\Lambda_i(t) = \Phi \sum_{\mathbf{j}} \mathbf{C}_{ij} \frac{I_{NVj}(t) + (1-VE) I_{Vi}(t)}{N_j} \tag{4}$$

The temporal dependence is induced by the variation in the number of infected across age brackets.

4.1.1 Derivation of R_0

A fundamental quantity in epidemiology is the basic reproductive number, R_0, defined as the number of secondary infections generated by a single infected individual in an otherwise susceptible population [10]. The basic reproductive number is a function of the disease's features and the contact patterns of the population. The next-generation matrix approach can be used to determine a closed-form expression for R_0.

$$R_0 = \frac{\Phi}{\Gamma} \rho(\tilde{\mathbf{C}}) \tag{5}$$

Where Φ is the transmissibility rate, Γ the recovery rate and $\rho(\tilde{\mathbf{C}})$ denotes the spectral radius of the matrix $\tilde{\mathbf{C}} = \frac{\mathbf{C}_{ij} N_i}{N_j}$.

4.2 Generalized models

We consider a SEIR model with vaccination featuring generalized contact matrices where the population is sliced in $m+1$ dimensions [6]. Age, plus m others dimensions. Here, we have considered $m = 1$, hence two dimensions in total. The epidemic dynamics are encoded in the following set of differential equations:

$$
\begin{aligned}
d_t S_{NV\mathbf{a}}(t) &= -\Lambda_{\mathbf{a}}(t) S_{NV\mathbf{a}}(t) - \Omega(t)_S, \\
d_t S_{V\mathbf{a}}(t) &= -(1-g_1)\Lambda_{\mathbf{a}}(t) S_{V\mathbf{a}}(t) + \Omega(t)_S, \\
d_t E_{NV\mathbf{a}}(t) &= \Lambda_{\mathbf{a}}(t) S_{NV\mathbf{a}}(t) - \Psi E_{NV\mathbf{a}}(t) - \Omega(t)_E, \\
d_t E_{V\mathbf{a}}(t) &= (1-g_1)\Lambda_{\mathbf{a}}(t) S_{V\mathbf{a}}(t) - \Psi E_{V\mathbf{a}}(t) + \Omega(t)_E, \\
d_t I_{NV\mathbf{a}}(t) &= \Psi E_{NV}(t) - \Gamma I_{NV\mathbf{a}}(t), \\
d_t I_{V\mathbf{a}}(t) &= \Psi E_{V\mathbf{a}}(t) - \Gamma I_{V\mathbf{a}}(t), \\
d_t R_{NV\mathbf{a}}(t) &= \Gamma I_{NV\mathbf{a}}(t) - \Omega(t)_R, \\
d_t R_{V\mathbf{a}}(t) &= \Gamma I_{V\mathbf{a}}(t) + \Omega(t)_R,
\end{aligned}
\tag{6}
$$

where $\mathbf{a} = (i, \alpha)$ is the index vector that describes the membership of individuals in the $m+1$ groups and the subscripts NV and V indicate respectively the non-vaccinated and vaccinated individual in each compartment. The force of infection can be written as:

$$
\Lambda_{\mathbf{a}}(t) = \Phi \sum_{\mathbf{b}} \mathbf{G}_{\mathbf{a},\mathbf{b}} \frac{I_{NV\mathbf{b}}(t) + (1-VE) I_{V\mathbf{b}}(t)}{N_{\mathbf{b}}} \tag{7}
$$

The temporal dependence is induced by the variation in the number of infected across age brackets.

4.2.1 Derivation of R_0

As shown in Ref. [6], the basic reproduction number R_0 for epidemic models that incorporate generalized contact matrices can be derived using the next-generation matrix approach, obtaining:

$$
R_0 = \frac{\Phi}{\Gamma} \rho(\tilde{\mathbf{G}}) \tag{8}
$$

As mentioned above, Φ is the transmissibility rate, Γ the recovery rate and $\rho(\tilde{\mathbf{G}})$ denotes the spectral radius of the matrix $\tilde{\mathbf{G}} = \frac{\mathbf{G}_{\mathbf{a}_\mathbf{b}} N_{\mathbf{a}}}{N_{\mathbf{b}}}$.

4.2.2 Modeling vaccination uptake among the second dimension

As shown in Fig A, vaccination data for Hungary on the daily doses administered is available, at the lower level, by age groups. To introduce a bias in the vaccination uptake among individuals in the second dimension, we use true data stratified by age and distribute it along the second dimension according to a given distribution $P_{vax}(\alpha)$, which indicates the proportion of vaccines allocated to each subgroup. Let $\Omega_i(t)$ represent the daily administered doses at time t for age group i. The number of vaccines administered to a subgroup α is then $\Omega_{i,\alpha}(t) = \Omega_i(t) \cdot P_{vax}(\alpha)$.

We assume that each subgroup can be vaccinated up to 95%. If this limit is reached, any excess vaccinations are redistributed randomly within that particular age group.

As detailed in the main text, we systematically compare four vaccination distributions along the second dimension (VD1–VD4), summarized in Table C.

5 Numerical simulations

We developed a stochastic, discrete-time, compartmental model, where transitions among compartments are simulated via chain binomial processes at each discrete time step $\Delta t = 1$ day. The population

Distribution	Low SES	Mid SES	High SES
VD1 (random)	0.35	0.45	0.20
VD2 (even)	$\frac{1}{3}$	$\frac{1}{3}$	$\frac{1}{3}$
VD3 (activity-based)	0.20	0.30	0.50
VD4 (real-world)	0.28	0.46	0.26

Table C: Vaccination distributions (VD) along the second dimension used in the simulations.

is divided into groups $\mathbf{a}$ (e.g., by age and socioeconomic status), and each compartment X (such as S, E, I, R, D) is updated according to probabilistic transitions derived from the model parameters.

At time step t, the number of individuals in group $\mathbf{a}$ moving from compartment X to compartment Y is sampled from a binomial distribution:

$$N_{X\mathbf{a}\to Y\mathbf{a}}(t) \sim PrBin\big(X_{\mathbf{a}}(t), p_{X_{\mathbf{a}}\to Y_{\mathbf{a}}}(t)\big), \tag{9}$$

where $PrBin(n, p)$ denotes a binomial distribution with n trials and success probability p. The probability $p_{X_{\mathbf{a}}\to Y_{\mathbf{a}}}(t)$ is computed based on the epidemiological parameters (e.g., force of infection $\Lambda_{\mathbf{a}}(t)$, recovery rate Γ, or progression rate Ψ).

For instance, the transition from susceptible (S) to exposed (E) individuals in group $\mathbf{a}$ is governed by:

$$p_{S_{\mathbf{a}}\to E_{\mathbf{a}}}(t) = 1 - \exp\big(-\Lambda_{\mathbf{a}}(t)\big) \tag{10}$$

where $\Lambda_{\mathbf{a}}(t)$ is the force of infection, computed as in equations (4) and (7). Similarly, the transition from exposed to infectious $p_{E_{\mathbf{a}}\to I_{\mathbf{a}}}(t) = \Psi$ and recovery or death transitions are defined via Γ and the infection fatality rate (IFR), respectively.

A single stochastic realization proceeds as follows:

1. Initialize compartments $S_{\mathbf{a}}(0)$, $E_{\mathbf{a}}(0)$, $I_{\mathbf{a}}(0)$, $R_{\mathbf{a}}(0)$, $D_{\mathbf{a}}(0)$.
2. For each time step $t = 1 \dots T$:
   (a) Compute the force of infection $\Lambda_{\mathbf{a}}(t)$ for all groups.
   (b) Sample new exposures: $S_{\mathbf{a}}(t) \to E_{\mathbf{a}}(t)$ via $PrBin(S_{\mathbf{a}}(t-1), p_{S_{\mathbf{a}}\to E_{\mathbf{a}}}(t))$.
   (c) Sample new infections: $E_{\mathbf{a}}(t) \to I_{\mathbf{a}}(t)$ via $PrBin(E_{\mathbf{a}}(t-1), \Psi)$.
   (d) Sample recoveries and deaths: $I_{\mathbf{a}}(t) \to R_{\mathbf{a}}(t)$ and $I_{\mathbf{a}}(t) \to D_{\mathbf{a}}(t)$ according to Γ and IFR.
   (e) Update vaccinated compartments based on $\Omega(t)$ (vaccination rate) and efficacy g_1, g_2.
3. Store compartment values at t and proceed to the next step.

This process is repeated for multiple stochastic realizations to account for variability in epidemic outcomes.

5.1 Pseudo-Code

Listing 1: Pseudocode of the SEIR model with vaccination

```
# Initialize model with epidemiological and vaccination parameters
initialize(model_type, vaccination_type, R0, mu, eps, IFR, VE, g1, g2, ...)

# Create compartments: S, E, I, R, D (and vaccinated versions)
init_compartments()

# Compute transmission rate phi from R0 and contact matrix C or Gab
compute_beta()

# Main time loop
for t = 1 to stop:
    if NPI and t >= T_npi:
```

```
        modify_contact_matrix(M_npi)

    for group in population_groups:
        lambda = compute_force_of_infection(group)
        new_E, new_I, new_R, new_D = transmission_step(group)
        update_compartments_transmission(group, new_E, new_I, new_R, new_D)

        Omega_t = compute_vaccination_DE(t)
        vaccination_dynamic(t, Omega_t)


return compartments_over_time
```

6 Computing attack rates

The attack rate (AR) is defined as the fraction of all individuals in a susceptible population who have been infected before a specific time t during an epidemic outbreak. In a compartmental SEIR model, the attack rate at time t can be computed as follows:

$$AR(t) = \frac{M_{S \to E}(t)}{N} \tag{11}$$

where $M_{(S \to E)}$ indicates the number of transitions from the susceptible (S) to the exposed (E) state up to time t, and N represents the total number of individuals in the population.

However, when vaccination is introduced, the computation of the attack rate for *vaccinated* and *non-vaccinated* individuals separately becomes more complex. In particular, when vaccination is administered during the course of the epidemic, not only are susceptible individuals vaccinated but also those who have been exposed (i.e., already infected but not yet infectious) and those who have recovered. In epidemiological modeling, this means that over the course of the epidemic, there are continuous movements between $S_{NV} \to S_V$ but also $E_{NV} \to E_V$ and $R_{NV} \to R_V$.

When computing the attack rate for *vaccinated* and *non-vaccinated* individuals separately, it is important to consider these transitions and account for the fact that vaccination is also administered to individuals in the E_{NV} and R_{NV} compartments. Therefore, the denominator of the attack rate is not simply the total number of vaccinated and non-vaccinated individuals at time t, but it should also account for these transitions.

Non-vaccinated population. For the non-vaccinated population, the denominator should include not only the total number of individuals not yet vaccinated at time t but also all the transitions from $E_{NV} \to E_V$ and $R_{NV} \to R_V$ up to time t. This is because these individuals, although vaccinated later, were not vaccinated when they were exposed or recovered. Thus, the adjusted denominator is:

$$N_{NV}(t) + M_{E_{NV} \to E_V}(t) + M_{R_{NV} \to R_V}(t) \tag{12}$$

Where $N_{NV}(t)$ is the total number of non-vaccinated individuals up to time t, and $M_{E_{NV} \to E_V}(t)$ and $M_{R_{NV} \to R_V}(t)$ indicate the number of individuals vaccinated up to time t in the exposed and recovered compartments, respectively. As $N_{NV}(t)$ can also be expressed as the difference between the total population and the vaccinated individuals, $N - N_V(t)$, and $N_V(t)$ can be expressed as the sum of all the vaccinated individuals, i.e., $M_{S_{NV} \to S_V}(t) + M_{E_{NV} \to E_V}(t) + M_{R_{NV} \to R_V}(t)$, we can rewrite Equation (12) as the difference between the total population and the number of individuals vaccinated in the susceptible compartment, the attack rate can be expressed as follow:

$$AR_{NV}(t) = \frac{M_{S_{NV} \to E_{NV}}(t)}{N - M_{S_{NV} \to S_V}(t)} \tag{13}$$

Vaccinated population. The denominator of the attack rate for vaccinated individuals should account for the fact that individuals moving from E_{NV} to E_V and R_{NV} to R_V were not vaccinated when they were initially infected. Therefore, these individuals should be removed from the count of the total number of vaccinated individuals at time t. Thus, the denominator becomes:

$$N_V(t) - M_{E_{NV}\to E_V}(t) - M_{R_{NV}\to R_V}(t) \tag{14}$$

By substituting $N_V(t)$ in Equation (14), we can compute the attack rate as follow:

$$AR_V(t) = \frac{M_{S_V\to E_V}(t)}{M_{S_{NV}\to S_V}(t)} \tag{15}$$

Entire population. Finally, the overall attack rate for the entire population can be computed as follows:

$$AR(t) = \frac{M_{S_{NV}\to E_{NV}}(t) + M_{S_V\to E_V}(t)}{N} \tag{16}$$

6.1 Estimating the attack rate of subgroups from age-stratified models

We define AR_α as the attack rate of subgroup α, that is, the fraction of individuals in subgroup α who have been infected by the end of the epidemic.

When adopting models that explicitly account for stratification of contacts across the dimension α computing AR_α is straightforward. In our case, contacts are stratified for age and a second dimension such as SES. Hence, AR_α can be obtained by aggregating the relevant quantities across all age groups within subgroup α.

In contrast, when the model is stratified by age only, additional steps are required to estimate, and approximate, SES-specific outputs, making the procedure more involved. In this section, we review how to estimate the attack rate for specific subgroups α (i.e., AR_α) using outputs from a model stratified solely by age. Note that in the main text, we refer to the result of this estimation as $f(\mathbf{C}_{ij})$. In other words, we refer to the procedure to estimate AR_α from the age-stratified models with the function f. As first step in the estimation process we need to infer the attack rate within each age group in the SES group α i.e., the subgroup (i, α). Subsequently we aggregate these age-specific results to obtain AR_α. This requires estimating the number of susceptible individuals in each group defined by age and SES (i, α) who became exposed during the epidemic ie., $M_{S_{i,\alpha} \to E_{i,\alpha}}$.

Assuming knowledge of the number of vaccinated individuals across subgroups defined by age and SES, one can infer this number using age-stratified output of traditional models. Namely, this involves computing the attack rate for both vaccinated and unvaccinated individuals, as described in equations (15) and (13) in each age group i, from the age-stratified model. Therefore, we can multiply AR_{NVi} and AR_{Vi} by the total number of non-vaccinated and vaccinated individuals in each group (i, α) at time t. However, in line with the considerations explained in the previous section, it is important to account for the transitions $E_{NVi} \to E_{Vi}$ and $R_{NVi} \to R_{Vi}$ that occur over the course of the epidemic. The equation used for this calculation reads as follows:

$$M_{S_{i,\alpha} \to E_{i,\alpha}} = AR_{Vi}(t) M_{S_{NVi,\alpha} \to S_{Vi,\alpha}}(t) + AR_{NVi}(t) \left(N_{i,\alpha} - M_{S_{NVi,\alpha} \to S_{Vi,\alpha}}(t) \right), \tag{17}$$

where $AR_{Vi}(t)$ and $AR_{NVi}(t)$ represent respectively the attack rates for vaccinated and non-vaccinated individuals in age group i and $M_{S_{NVi,\alpha} \to S_{Vi,\alpha}}(t)$ denotes the number of susceptible individuals in groups (i, α) who were vaccinated at time t. In principle, we can estimate the number of vaccines administered to those who have already recovered from the infection, especially if they were symptomatic. However, it is not possible to determine the number of vaccines administered to individuals who were exposed. The attack rate for groups (i, α) can be then calculated by dividing equation (17) for the population size as follows:

$$AR_{i,\alpha} = \frac{AR_{Vi}(t) M_{S_{NVi,\alpha} \to S_{Vi,\alpha}}(t) + AR_{NVi}(t) \left(N_{i,\alpha} - M_{S_{NVi,\alpha} \to S_{Vi,\alpha}}(t) \right)}{N_{i,\alpha}} \tag{18}$$

Finally, we can then aggregate by age groups equation (18) and divide by the total number of individuals in subgroup α as follows:

$$AR_\alpha = \frac{\sum_i \left[AR_{Vi}(t) M_{S_{NVi\alpha} \to S_{Vi\alpha}}(t) + AR_{NVi}(t) \left(N_{i,\alpha} - M_{S_{NVi\alpha} \to S_{Vi\alpha}}(t) \right) \right]}{N_\alpha} \tag{19}$$

7 Robustness analysis for Figs 1 and 3

In this section, we present a series of alternative simulation settings for Figs 1 and 3 of the main text to assess the robustness of the results. By exploring different assumptions and parameters, we aim to ensure that the findings remain consistent across a range of plausible scenarios. These robustness checks provide additional confidence in the conclusions drawn from the main simulations.

7.1 Assortative mixing regime

7.1.1 Vaccination stratified only by *dim2*

Keeping the same simulation setting presented in the main, here we simulate vaccination distributions that do not account for age by only stratifying the vaccination distributions $VD1$, $VD2$, $VD3$ and $VD4$ across three groups of the second dimension (i.e., *dim2*). This approach is applied to both Scenario 1 and Scenario 2, as shown respectively in Figs D and E.

Scenario 1. In these simulations, the differences among the four vaccination distributions (VDs) are more pronounced in terms of overall prevalence across the population, as shown in panel *a* of Fig D. Fig D*B* displays the percentage of vaccinated individuals in each group along *dim2*, under the various vaccination distributions. Fig D*C* presents the attack rate per one thousand individuals (i.e., the number of cases per 1000 people) for each subgroup and vaccination distribution. Differences in attack rates among SES subgroups attack persist under this vaccination regimen as well. Finally, Fig D*D* demonstrates that the attack rates for groups along *dim2* are not accurately predicted by the age-stratified model. Specifically, the discrepancy between the two methods, $\mathbf{G_{ab}} - f(\mathbf{C}_{ij})$, is always non-zero.

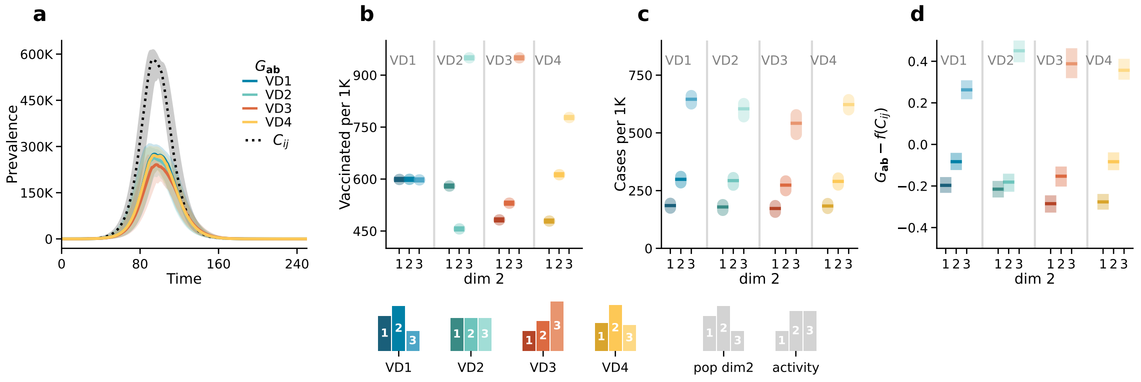

Figure D: **Scenario 1. Epidemic outcomes and predictability**: Panel *a* displays the number of newly infected individuals over time. The dotted black line corresponds to the outcome of the age-stratified model (i.e., $\mathbf{C}_{ij}$), and the solid coloured lines correspond to the outcome of the generalized model (i.e., $\mathbf{G_{ab}}$) in the three different vaccination strategies ($VD1, VD2, VD3, VD4$). Panel *b* shows the percentage of vaccinated individuals in the three subgroups (i.e., *dim2*). Panel *c* shows the attack rate by the second dimension predicted by the generalized model. Panel *d* shows the difference between the attack rate predicted by the generalized model and the one estimated from the aggregate output of the age-stratified model (i.e., $f(\mathbf{C}_{ij})$) by the second dimension. Results refer to the median of 500 runs with IQRs (shaded area). Epidemiological parameters: $\Gamma = 0.25, \Psi = 0.4, g_1 = 0.6, R_0 = 2$. Simulations start with $I_0 = 100$ initial infectious seeds.

Scenario 2. Fig G presents the same analysis for Scenario 2. Interestingly, Fig G*a* and Fig G *c* reveal a different pattern in terms of overall prevalence and attack rate across vaccination distributions. In this case, only $VD1$ (random distribution of vaccines) allows the epidemic to take off, while all other vaccination strategies effectively prevent the spread from the outset.

Overall, these simulation results align with the findings discussed in the main text, supporting the conclusions on the influence of vaccination distribution patterns on epidemic outcomes.

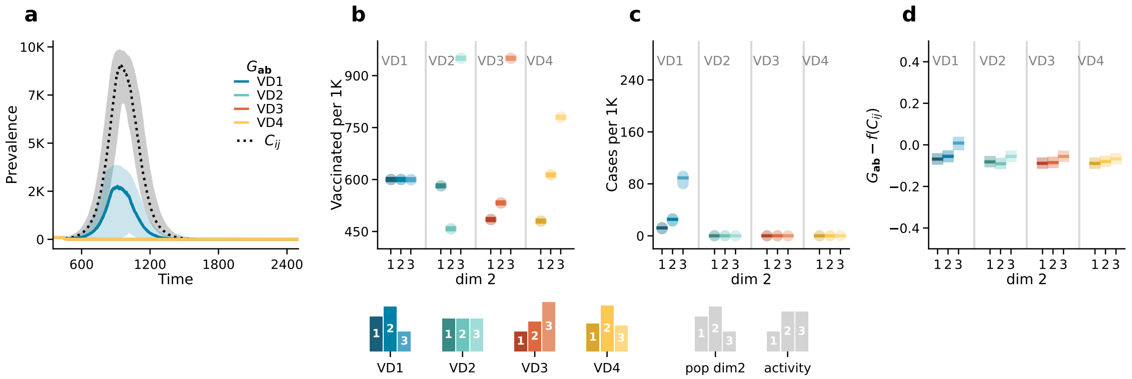

Figure E: **Scenario 2. Epidemic outcomes and predictability**: Panel *a* displays the number of newly infected individuals over time. The dotted black line corresponds to the outcome of the age-stratified model (i.e., $\mathbf{C}_{ij}$), and the solid coloured lines correspond to the outcome of the generalized model (i.e., $\mathbf{G_{ab}}$) in the three different vaccination strategies ($VD1, VD2, VD3, VD4$). Panel *b* shows the percentage of vaccinated individuals in the three subgroups (i.e., *dim2*). Panel *c* shows the attack rate by the second dimension predicted by the generalized model. Panel *d* shows the difference between the attack rate predicted by the generalized model and the one estimated from the aggregate output of the age-stratified model (i.e., $f(\mathbf{C}_{ij})$) by the second dimension. Results refer to the median of 500 runs with IQRs (shaded area). Epidemiological parameters: $\Gamma = 0.25, \Psi = 0.4,\ g_1 = 0.6,\ R_0 = 2$. Simulations start with $I_0 = 100$ initial infectious seeds.

7.2 Random mixing regime

In this section, we replicate the analysis presented in Fig 1 (Scenario 1) and Fig 3 (Scenario 2) of the main text, using a simplified population setting with equal share across the three groups and random mixing. In doing so, we first consider a scenario where the vaccination is stratified by age and *dim2*. Then, we consider a stratification of vaccines only along the *dim2* dimension.

We assume a random mixing where contacts along the second dimension are set proportional to the product of the population sizes in each group as explained above in Section 2.2. Hence, the only set of parameters needed are those regulating the population distribution, which is set to $\frac{1}{3}, \frac{1}{3}, \frac{1}{3}$.

7.2.1 Vaccination stratified by age and *dim2*

Scenario 1. The first observation is that, as expected from Ref. [6], homogeneous mixing leads to the same epidemic dynamics (i.e., same prevalence) across subgroups (see Fig FA). Indeed, regardless of the vaccination distribution, the colored lines overlap. Furthermore, random mixing leads to the same total prevalence regardless of the inclusion of a second dimension in the contact matrices, as also discussed in Ref. [6] Indeed, solid colored and dashed grey lines representing, respectively, the prevalence estimated by modes fed with generalized or traditional contact matrices overlap. In Fig Fb, we show the percentage of vaccinated individuals in each group along *dim2*, under different vaccination distributions (VDs). It is important to note that in a random mixing scenario, the interpretation of these distributions changes. $VD2$ corresponds to a uniform allocation of vaccines across the second dimension, while $VD1$, $VD3$, and $VD4$ represent preferential distributions, where vaccine coverage varies across *dim2*. In Fig FC, we show the attack rate per one thousand (i.e., number of cases for 1000 individuals) for each subgroup, for each VDs. In this situation, differences in disease burden among subgroups arise solely from variations in vaccination uptake. When vaccination uptake is equal across all groups ($VD2$), infection rates are the same in all three SES groups. Under vaccination distribution $VD2$ and $VD4$, the second SES group shows the lowest infection rate due to higher vaccination uptake. In the more unequal distribution of vaccines ($VD3$), the number of cases in each group inversely correlates with their respective vaccination uptake: the first group, having the lowest vaccination rate, becomes the most infected, while the third group, with the highest vaccination uptake, experiences the fewest cases. Finally, in Fig HD we show the difference of the number of cases in each subgroup from the generalized model $\mathbf{G_{ab}}$ and as estimated from a simple age-stratified model $f(\mathbf{C}_{ij})$. In this scenario, the differences among the two techniques, although small, are almost always different from zero.

Scenario 2. Here, we report the results of the same simulation setting for Scenario 2, where the vaccination campaign rollout happened before the epidemic outbreak.

Similarly to Scenario 1, panel a of Fig GA shows that the colored lines overlap regardless of the vaccination distribution, indicating consistent epidemic outcomes across different distribution strategies. Fig GB, we show the percentage of vaccinated individuals in each group along *dim2*, under different vaccination distributions (VDs). Fig GC shows the attack rate per one thousand (i.e., number of cases for 1000 individuals) for each subgroup, for each vaccination distribution (VDs). As before, in a random mixing situation, differences in disease burden among subgroups arise solely from variations in vaccination uptake. Thus, the qualitative differences among the attack rates in subgroups follow the ones seen in Scenario 1. Fig GD shows the difference of the number of cases in each subgroup from the generalized model $\mathbf{G_{ab}}$ and as estimated from a simple age-stratified model $f(\mathbf{C}_{ij})$.

Interestingly, the difference is always zero, meaning that, in this case, it is possible to accurately predict the attack rate in the three additional subgroups starting from the output from age-stratified models. Indeed, in this scenario, since the vaccination is already completed before the epidemic begins, there is not a dynamic interaction between the vaccination uptake and the epidemic spreading, thus the attack rate in the different subgroups is fully captured by the overall number of individuals in each subgroup, that were vaccinated before the epidemic started.

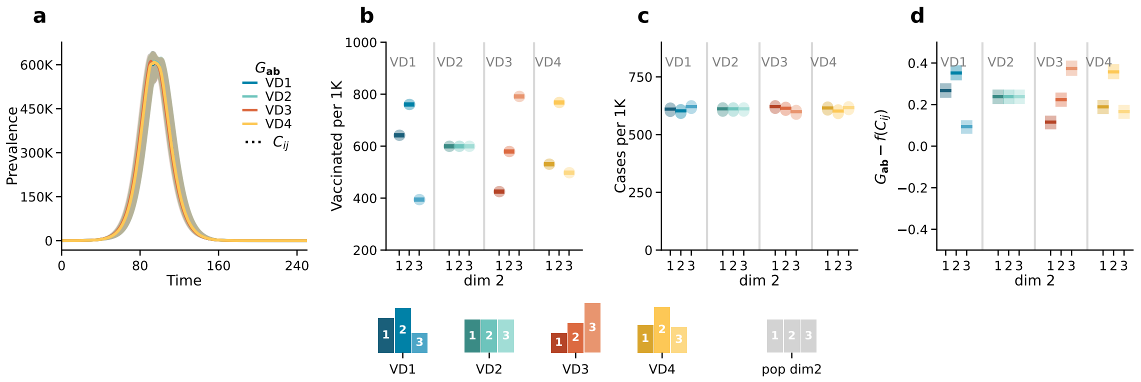

Figure F: **Scenario 1. Epidemic outcomes and predictability**: Panel *a* displays the number of newly infected individuals over time. The dotted black line corresponds to the outcome of the age-stratified model (i.e., $\mathbf{C}_{ij}$), and the solid coloured lines correspond to the outcome of the generalized model (i.e., $\mathbf{G_{ab}}$) in the three different vaccination strategies (i.e., $VD1, VD2, VD3, VD4$). Panel *b* shows the percentage of vaccinated individuals in the three subgroups (i.e., *dim2*). Panel *c* shows the attack rate by the second dimension predicted by the generalized model. Panel *d* shows the difference between the attack rate predicted by the generalized model and the one estimated from the aggregate output of the age-stratified model (i.e., $f(\mathbf{C}_{ij})$) by the second dimension. Results refer to the median of 500 runs with IQRs (shaded area). Epidemiological parameters: $\Gamma = 0.25, \Psi = 0.4,\ g_1 = 0.6,\ R_0 = 2$. Simulations start with $I_0 = 100$ initial infectious seeds.

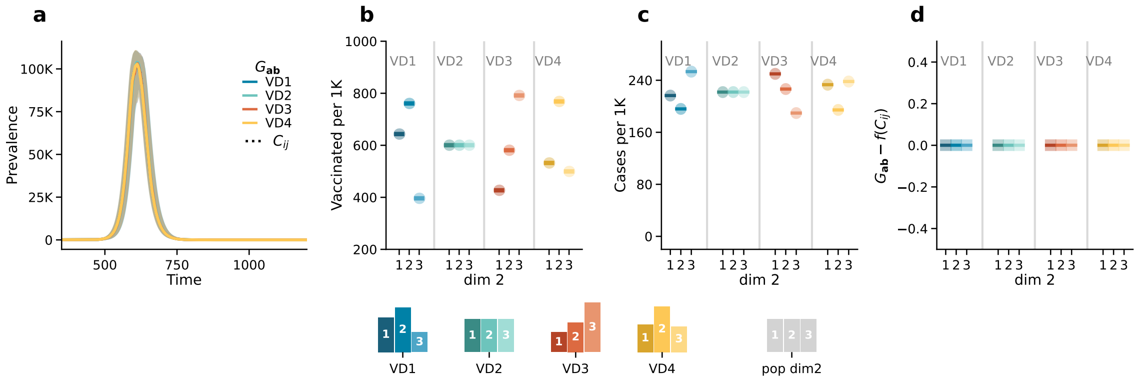

Figure G: **Scenario 2. Epidemic outcomes and predictability**: Panel *a* displays the number of newly infected individuals over time. The dotted black line corresponds to the outcome of the age-stratified model (i.e., $\mathbf{C}_{ij}$), and the solid colored lines correspond to the outcome of the generalized model (i.e., $\mathbf{G_{ab}}$) in the three different vaccination strategies ($VD1, VD2, VD3, VD4$). Panel *b* shows the percentage of vaccinated individuals in the three subgroups (i.e., *dim2*). Panel *c* shows the attack rate by the second dimension predicted by the generalized model. Panel *d* shows the difference between the attack rate predicted by the generalized model and the one estimated from the aggregate output of the age-stratified model (i.e., $f(\mathbf{C}_{ij})$) by the second dimension. Results refer to the median of 500 runs with IQRs (shaded area). Epidemiological parameters: $\Gamma = 0.25, \Psi = 0.4,\ g_1 = 0.6,\ R_0 = 2$. Simulations start with $I_0 = 100$ initial infectious seeds.

7.2.2 Vaccination stratified only by *dim2*

We also report the results obtained when the vaccination is stratified only by SES. As mentioned above, also in this scenario we consider random mixing. The qualitative outcomes are consistent with those discussed in the previous section. The epidemic dynamics remain unaffected by the choice of vaccination distribution due to the absence of structured mixing, and differences in disease burden among SES groups arise solely from differences in vaccine uptake. In particular, in Scenario 2 in Fig H*D*, we observe that the generalized model and the aggregate output from the age-stratified ($f(\mathbf{C}_{ij})$) model yield identical predictions, confirming that in the absence of dynamic feedback between vaccination and transmission, in a random mixing setting, the attack rate can be accurately inferred from aggregate vaccination coverage.

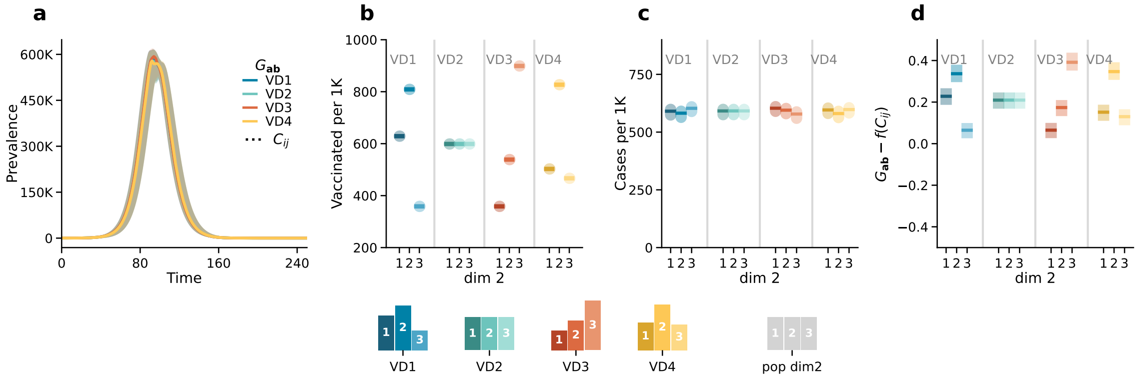

Figure H: **Scenario 1. Epidemic outcomes and predictability**: Panel *a* displays the number of newly infected individuals over time. The dotted black line corresponds to the outcome of the age-stratified model (i.e., $\mathbf{C}_{ij}$), and the solid coloured lines correspond to the outcome of the generalized model (i.e., $\mathbf{G_{ab}}$) in the three different vaccination strategies (i.e., $VD1, VD2, VD3, VD4$). Panel *b* shows the percentage of vaccinated individuals in the three subgroups (i.e., *dim2*). Panel *c* shows the attack rate by the second dimension predicted by the generalized model. Panel *d* shows the difference between the attack rate predicted by the generalized model and the one estimated from the aggregate output of the age-stratified model (i.e., $f(\mathbf{C}_{ij})$) by the second dimension. Results refer to the median of 500 runs with IQRs (shaded area). Epidemiological parameters: $\Gamma = 0.25, \Psi = 0.4$, $g_1 = 0.6$, $R_0 = 2$. Simulations start with $I_0 = 100$ initial infectious seeds.

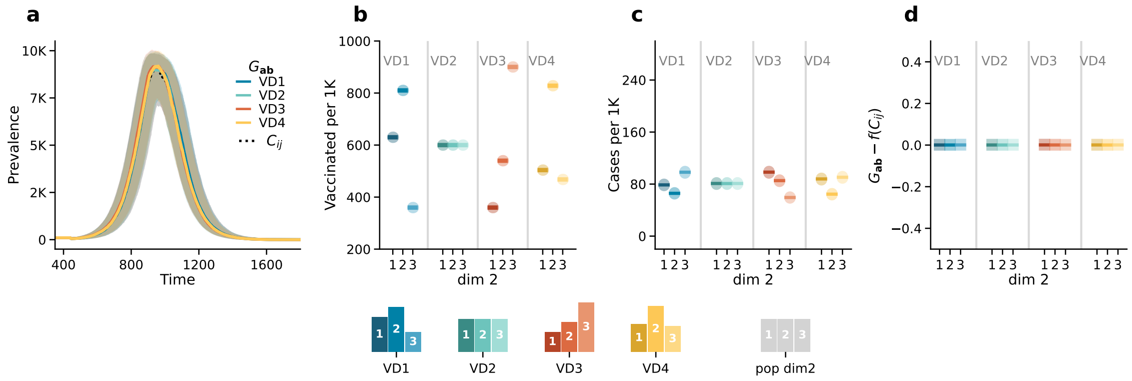

Figure I: **Scenario 2. Epidemic outcomes and predictability**: Panel a displays the number of newly infected individuals over time. The dotted black line corresponds to the outcome of the age-stratified model (i.e., $\mathbf{C}_{ij}$), and the solid colored lines correspond to the outcome of the generalized model (i.e., $\mathbf{G_{ab}}$) in the three different vaccination strategies (i.e., $VD1, VD2, VD3, VD4$). Panel b shows the percentage of vaccinated individuals in the three subgroups (i.e., *dim2*). Panel c shows the attack rate by the second dimension predicted by the generalized model. Panel d shows the difference between the attack rate predicted by the generalized model and the one estimated from the aggregate output of the age-stratified model (i.e., $f(\mathbf{C}_{ij})$) by the second dimension. Results refer to the median of 500 runs with IQRs (shaded area). Epidemiological parameters: $\Gamma = 0.25, \Psi = 0.4$, $g_1 = 0.6$, $R_0 = 2$. Simulations start with $I_0 = 100$ initial infectious seeds.

7.3 Sensitivity to varying t_{epi} and R_0

Finally, we run a sensitivity analysis for the difference between the number of cases in each subgroup obtained from the generalized model and as estimated from a simple age-stratified model (i.e., $\mathbf{G_{ab}} - f(\mathbf{C}_{ij})$).

In Figs J and K, we show how this difference changes (i) as a function of t_{epi}, the time at which the epidemic starts given that vaccination began at $t = 0$ with $R_0 = 2$, and (ii) as a function of R_0, the reproduction number, with $t_{epi} = 0$.

Both the parameters t_{epi} and R_0 determine the overlap between the vaccination campaign and the epidemiological wave. Indeed, with high values of t_{epi}, vaccination might be completed before the epidemic starts, whereas with high values of R_0, the epidemic might peak early during the vaccination campaign, diminishing the vaccination's impact on the epidemic curve. We run this analysis using the same epidemic parameters as in the main text, except for the parameters varied in the sensitivity analysis (i.e., t_{epi} and R_0). We explore the effect of different vaccination strategies under two mixing regimes:

- **Vaccination stratified by both age and *dim2*** (as in the main text) (Fig J),
- **Vaccination stratified only by the second dimension (i.e., *dim2*)** (Fig K).

For each vaccination strategy, we simulate both a random mixing scenario with equal population distributions across the three groups (see panel a) and an assortative mixing scenario and population distribution as in Table D (see panel b).

7.3.1 Vaccination stratified by age and *dim2*

We begin by analyzing the case in which vaccination is stratified by both age and a second dimension (*dim2*). The results are presented in Fig J. Interestingly, in this scenario, under random mixing, where vaccination is completed before the epidemic begins, we can accurately predict the attack rate within each subgroup based on the output of an age-stratified model. Specifically, in J*a* first column, we can observe that the difference $(\mathbf{G_{ab}} - f(\mathbf{C}_{ij}))$ converges to zero as t_{epi} increases, indicating that in a fully vaccinated population with no interaction between epidemic and vaccination dynamics, the attack rate by subgroup aligns with predictions from the age-stratified model. However, as the overlap between the vaccination campaign and the epidemic increases, so does the error, as shown in panel a, first column, for low values of t_{epi} and in the second column for higher values of R_0. In contrast, under assortative mixing, the interaction between differences in individual activity levels and vaccination uptake prevents accurate predictions (panel b).

The transient and non-linear behavior observed in panel b emerges from the interplay between the epidemic dynamics and the timing of vaccination, which is modulated by both R_0 and t_{epi}. When $t_{epi} = 0$, increasing R_0 leads to shorter epidemic durations, thereby reducing the final fraction of individuals who are vaccinated before exposure, and resulting in abrupt transitions in the attack rate differences. This non-linearity reflects a shift between slow and fast epidemic regimes. Conversely, when R_0 is fixed (e.g., $R_0 = 2$), increasing t_{epi} allows more individuals to be vaccinated before the epidemic begins, leading to a gradual reduction in the discrepancy until it saturates.

7.3.2 Vaccination stratified only by *dim2*

We then focus on a simplified vaccination strategy in which vaccine allocation is stratified only by the second dimension (*dim2*), regardless of age. We show the results in Fig K. As before, we examine both a homogeneous mixing setting (panel A) and an assortative mixing setting (panel B). Consistent patterns emerge, leading to the same qualitative results and outcomes. Indeed, results indicate that under random mixing, predictions of subgroup attack rates align closely with those from an age-stratified model when vaccination concludes prior to the epidemic onset. However, as the overlap between the vaccination campaign and the epidemic grows, discrepancies increase. This effect is particularly pronounced under assortative mixing, where the interaction between subgroup activity differences and vaccination uptake prevents accurate predictions.

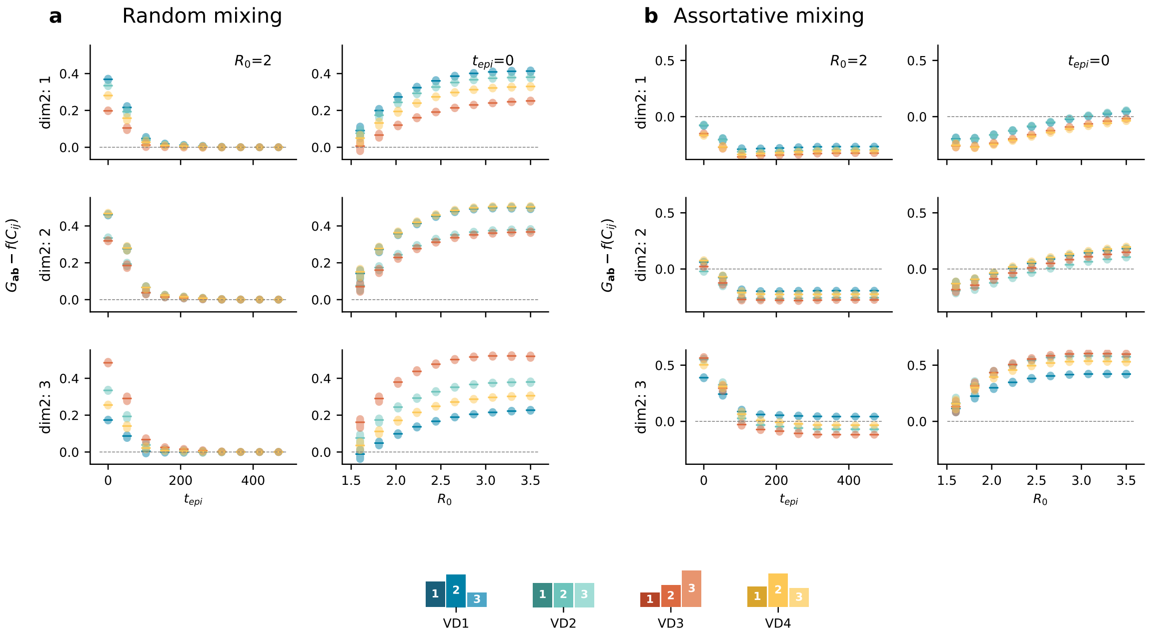

Figure J: **Sensitivity analysis to varying t_{epi} and R_0.** Differences between the overall attack rate for each subgroup as predicted by the generalized model $\mathbf{G}_{ab}$ and the attack rate predicted by the aggregate output of the age-stratified model, $f(\mathbf{C}_{ij})$ as a function of (i) t_{epi} with $R_0 = 2$, and (ii) R_0 with $t_{epi} = 0$. These analyses are done both under a random mixing setting (panel a) and an assortative mixing setting (panel b). Results refer to the median of 500 runs with IQRs (shaded area).

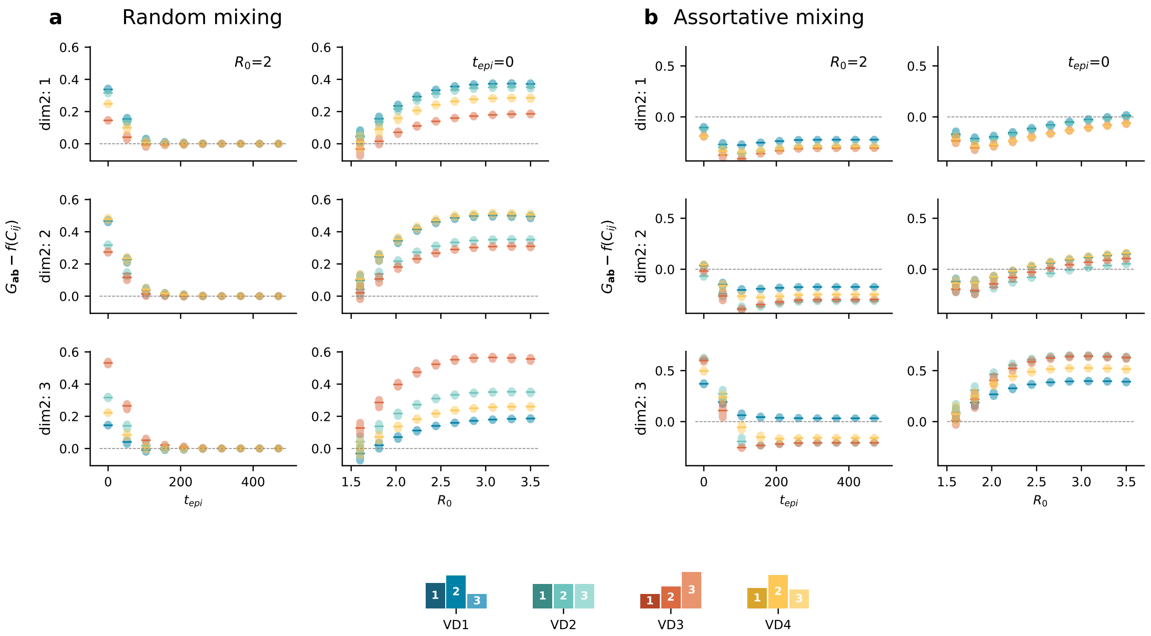

Figure K: **Sensitivity analysis to varying t_{epi} and R_0.** Differences between the overall attack rate for each subgroup as predicted by the generalized model $\mathbf{G}_{ab}$ and the attack rate predicted by the aggregate output of the age-stratified model, $f(\mathbf{C}_{ij})$ as a function of (i) t_{epi} with $R_0 = 2$, and (ii) R_0 with $t_{epi} = 0$. These analysis are done both under a random mixing setting (panel a) and an assortative mixing setting (panel b). Results refer to the median of 500 runs with IQRs (shaded area).

8 Non pharmaceutical interventions

Table D reports the values for activity, assortativity, and other free parameters used for both the tightened NPI simulation presented in Fig 2 (Scenario 1) and the relaxed NPI simulation presented in Fig 4 (Scenario 2) of the main text.

SES Group	**1**	**2**	**3**
Assortativity	50%	60%	70%
Activity	40%	25%	35%
Free parameters	0.5	0.4	0.4

Table D: List of parameters used to simulate the NPIs

For completeness, we extend the model by adding a new compartment (D) to estimate the number of deaths under each vaccination distribution. In particular, we simulate the number of daily deaths by applying the age-stratified Infection Fatality Rate (IFR_i) estimated for COVID-19 by Ref.[11] and detailed in Table E.

**Age group**	IFR_i
[0–5)	0.00001
[5–15)	0.00001
[15–30)	0.00005
[30–45)	0.0002
[45–60)	0.002
[60–70)	0.007
[70–80)	0.019
[80+)	0.083

Table E: Infection Fatality Rate by age group

Therefore, to model death, the set of the equations presented in (6) can be extended as follows:

$$\begin{aligned}
d_t S_{NV\mathbf{a}}(t) &= -\Lambda_{\mathbf{a}}(t) S_{NV\mathbf{a}}(t) - \Omega(t)_S, \\
d_t S_{V\mathbf{a}}(t) &= -(1-g_1)\Lambda_{\mathbf{a}}(t) S_{V\mathbf{a}}(t) + \Omega(t)_S, \\
d_t E_{NV\mathbf{a}}(t) &= \Lambda_{\mathbf{a}}(t) S_{NV\mathbf{a}}(t) - \Psi E_{NV\mathbf{a}}(t) - \Omega(t)_E, \\
d_t E_{V\mathbf{a}}(t) &= (1-g_1)\Lambda_{\mathbf{a}}(t) S_{V\mathbf{a}}(t) - \Psi E_{V\mathbf{a}}(t) + \Omega(t)_E, \\
d_t I_{NV\mathbf{a}}(t) &= \Psi E_{NV}(t) - \Gamma I_{NV\mathbf{a}}(t), \\
d_t I_{V\mathbf{a}}(t) &= \Psi E_{V\mathbf{a}}(t) - \Gamma I_{V\mathbf{a}}(t), \\
d_t R_{NV\mathbf{a}}(t) &= (1 - IFR_i)\Gamma I_{NV\mathbf{a}}(t) - \Omega(t)_R, \\
d_t R_{V\mathbf{a}}(t) &= (1-(1-g_2)IFR_i))\Gamma I_{V\mathbf{a}}(t) + \Omega(t)_R, \\
d_t D_{NV\mathbf{a}}(t) &= IFR_i \Gamma I_{NV\mathbf{a}}(t), \\
d_t D_{V\mathbf{a}}(t) &= (1 - IFR_i)\Gamma I_{V\mathbf{a}}(t)
\end{aligned} \tag{20}$$

Section 1. We report the attack rate by socioeconomic status (SES) for both the Baseline setting and the setting with non-pharmaceutical interventions (NPI) in Fig L.

Fig M presents the results of the model with deaths for Scenario 1. In particular, panels a and b show respectively the prevalence in the baseline case and the case with tightened NPIs where contacts are reduced by 20%. Similarly, panels c and d display the total number of deaths in the baseline case and under tightened NPIs, respectively. The mortality trend exhibits the same qualitative behaviour as the prevalence.

Scenario 2. Similarly, we report the attack rate by socioeconomic status (SES) for both the Baseline setting and the setting with non-pharmaceutical interventions (NPI) in Fig N.

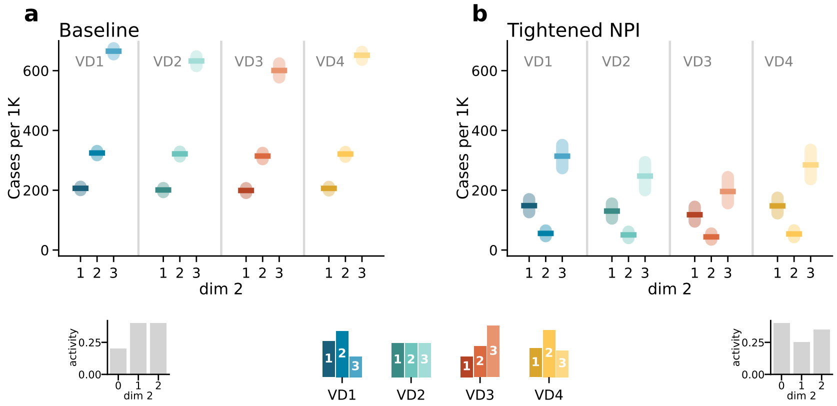

Figure L: **Scenario 1: The impact of NPIs on SES groups under different vaccination distributions.** Panel a refers to the baseline case and shows the attack rate scaled by 1000 for the three SES. Panel b follows the same structure but refers to the case where tightened NPIs are introduced, reducing contacts by 20%. NPIs are adjusted 65 days after the epidemic onset. Results represent the median of 500 runs with confidence intervals. Epidemiological parameters: $\Gamma = 0.25$, $\Psi = 0.4$, $g_1 = 0.6$, $g_2 = 0.8$, and $R_0 = 2$. Simulations begin with $I_0 = 100$ initial infected seeds.

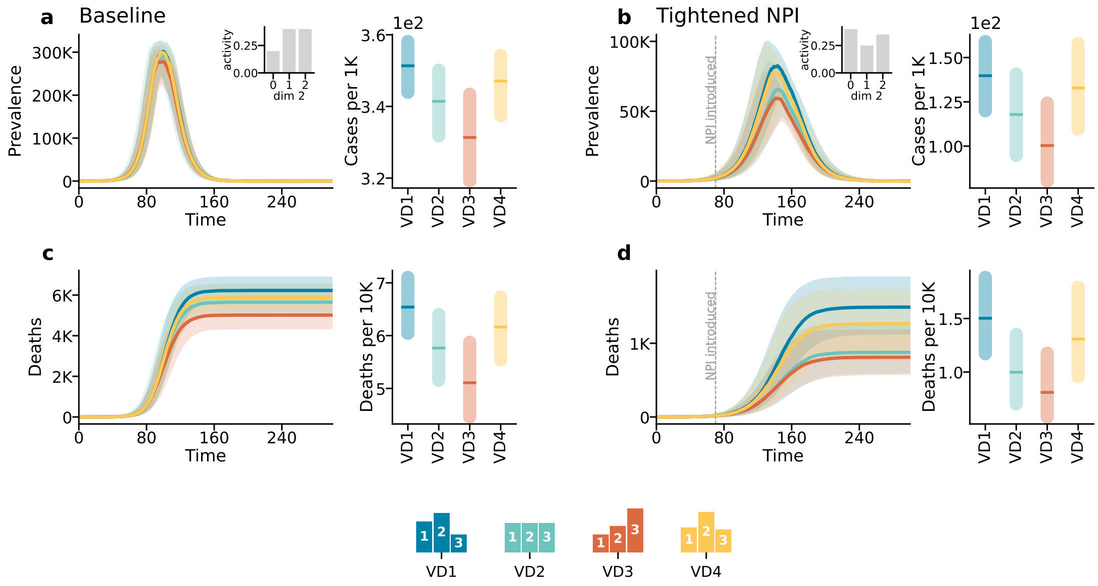

Figure M: **Scenario 1: The impact of NPIs under different vaccination distributions.** Panel a refers to the baseline case, with insets depicting the activity distribution along the second dimension. The first column shows the total number of infected individuals, while the second column shows the attack rate scaled by 1000. Panel b follows the same structure but refers to the case where tightened NPIs are introduced, reducing contacts by 20%. Panels c and d follow the same structure but refer to the total number of deaths (first column) and mortality rate scaled by $10K$ (second column). NPIs are adjusted 65 days after the epidemic onset. Results refer to the median of 500 runs with IQRs (shaded area). Epidemiological parameters: $\Gamma = 0.25$, $\Psi = 0.4$, $g_1 = 0.6$, $g_2 = 0.8$, and $R_0 = 2$. Simulations begin with $I_0 = 100$ initial infected seeds.

While Fig O presents the results of the model that includes mortality for Scenario 2 following the same structure of Fig M. Also in this case the mortality trend exhibits the same qualitative behavior as the prevalence.

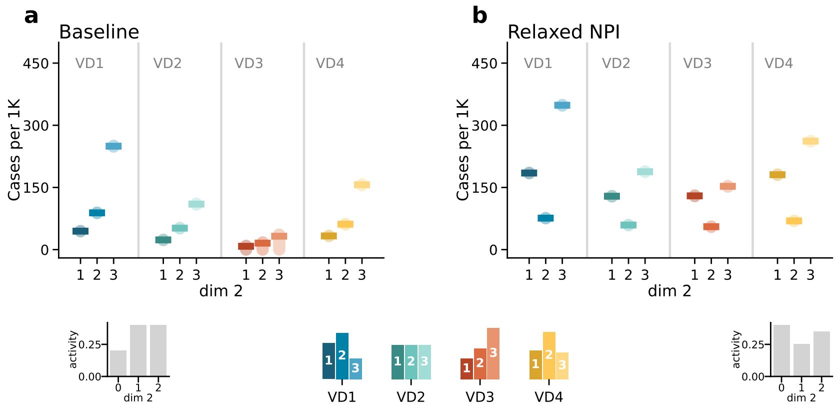

Figure N: **Scenario 2: The impact of NPIs on SES groups under different vaccination distributions.** Panel *a* refers to the baseline case and shows the attack rate scaled by 1000 for the three SES. Panel *b* follows the same structure but refers to the case where tightened NPIs are released and the contacts increase by 20%. Panels *c* and *d* follow the same structure but refer to the total number of deaths (first column) and mortality rate scaled by $10K$ (second column). NPIs are adjusted 65 days after the epidemic onset. Results refer to the median of 500 runs with IQRs (shaded area). Epidemiological parameters: $\Gamma = 0.25$, $\Psi = 0.4$, $g_1 = 0.6$, $g_2 = 0.8$, and $R_0 = 2$. Simulations begin with $I_0 = 100$ initial infected seeds.

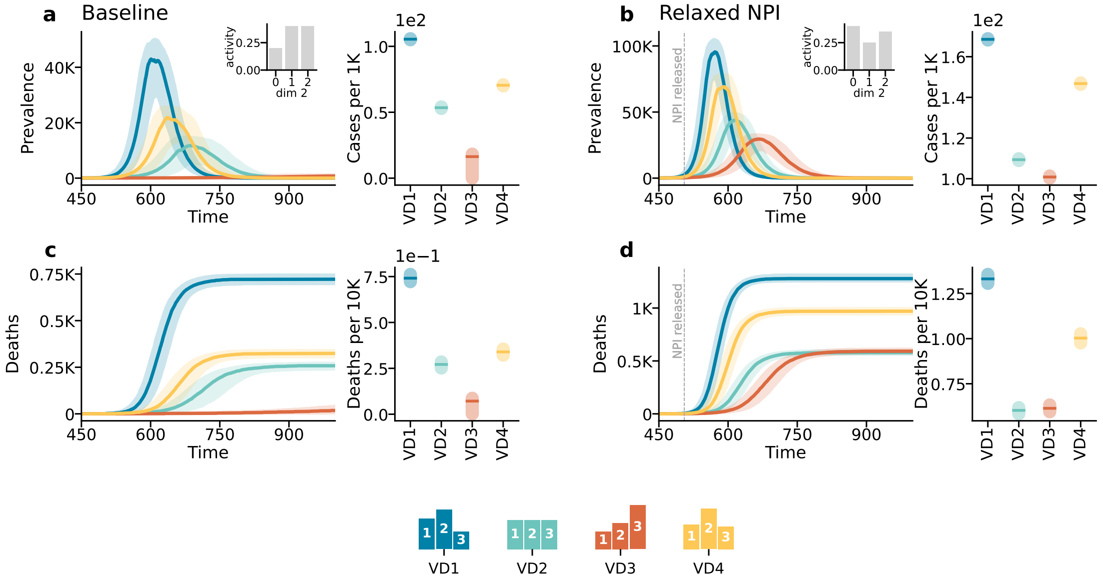

Figure O: **Scenario 2: The impact of NPIs under different vaccination distributions.** Panel *a* refers to the baseline case, with insets depicting the activity distribution along the second dimension. The first column shows the total number of infected individuals, while the second column shows the attack rate scaled by 1000. Panel *b* follows the same structure but refers to the case where tightened NPIs are released, increasing contacts by 20%. NPIs are adjusted 65 days after the epidemic onset. Results refer to the median of 500 runs with IQRs (shaded area). Epidemiological parameters: $\Gamma = 0.25$, $\Psi = 0.4$, $g_1 = 0.6$, $g_2 = 0.8$, and $R_0 = 2$. Simulations begin with $I_0 = 100$ initial infected seeds.

9 Hungarian contact data

In Fig 5 of the main text, we use data from the MASZK project to derive real-world generalized contact matrices for Hungary. This dataset provides information on social interactions in two formats: aggregate reports of contact counts by age group, and detailed diaries documenting individual contacts with associated age and SES attributes. While diary data are available only for adults (aged 15+), aggregate data for children include SES information only for the respondent, not for their contacts. To infer SES-specific contact patterns among children, we assumed assortative mixing along SES and introduced a redistribution matrix $u_{\alpha\beta}$ that allocates contacts across SES groups. The values of $u_{\alpha\beta}$ are set as follows:

$$u_{\alpha\beta} = \begin{pmatrix} 0.7 & 0.2 & 0.1 \\ 0.1 & 0.7 & 0.2 \\ 0.1 & 0.2 & 0.7 \end{pmatrix} \tag{21}$$

The resulting contact matrix $\mathbf{G}_{\mathbf{a},\mathbf{b}}$, where $\mathbf{a} = (i, \alpha)$ and $\mathbf{b} = (j, \beta)$, reflects these assumptions. The matrices also incorporate household contacts, assigned under the assumption that family members share the same SES. For more details on the methodology used to build these matrices, see Ref. [6].

9.1 Assortativity by SES

In this section, we use the real-world generalized contact matrices described above to explore individuals' assortativity by socioeconomic status (SES) across different age groups—that is, how likely individuals of different age groups are to interact with others from the same SES. We report the SES assortativity values for each age group and SES, during the 2nd and 4th waves in Hungary (corresponding to Scenario 1 and Scenario 2, respectively). The SES assortativity for age group i and SES α ($A_{i,\alpha}$) is computed as follows:

$$A_{i,\alpha} = \frac{\sum_j \mathbf{G}_{i\alpha,j\alpha}}{\sum_{j,\beta} \mathbf{G}_{i\alpha,j\beta}} \tag{22}$$

Where, $\mathbf{G}_{i\alpha,j\beta}$ is the contact rate between individuals in age group i and SES α with individuals in age group j and SES β. In Fig P we show the age-specific assortativity by socioeconomic status (SES) computed for Scenario 1 and 2. We note that individuals experiencing high SES are indeed the most assortative, followed by those experiencing low SES. Aditionally, we observe how assortativity generally decreases, across SES, with age.

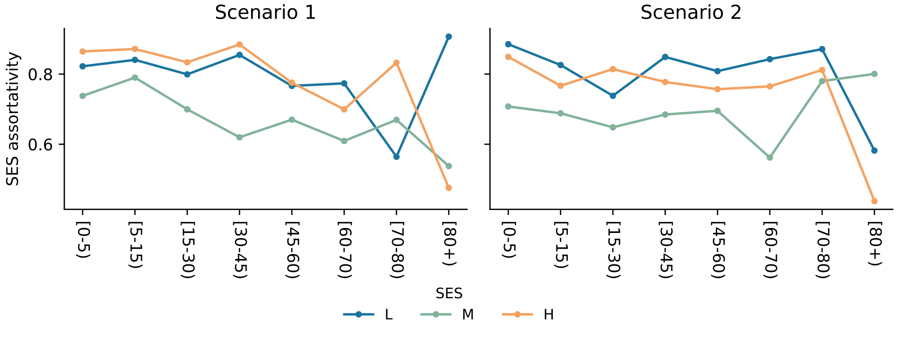

Figure P: **Age-specific assortativity by socioeconomic status (SES)**. For each age group and SES level (low: L, medium: M, high: H), the index measures the proportion of contacts individuals have with others of the same SES group. Higher values indicate stronger assortative mixing by SES. Results are shown for Scenario 1 (left) and Scenario 2 (right), based on contact matrices stratified by age and SES.

9.2 Vaccination distributions using real-world contact matrices

When using real-world generalized contact matrices (Fig 5 in main text), we apply the same vaccination strategies as in the synthetic scenarios. However, these strategies do not account for the correlation between socioeconomic status (SES) and age present in the empirical data. In reality, the age distribution varies across SES groups (e.g., lower SES groups tend to include younger individuals), while the vaccination strategies prioritize age uniformly across all SES groups. As a result, the SES distribution of vaccinated individuals in the real-data scenarios does not match that of the synthetic setting. For example, the strategy $VD1$, which we refer to as "proportional to group sizes (i.e., random distribution)", does not produce a uniform distribution across SES groups in the empirical case. To emphasize this, we marked the VD symbol with an asterisk. Additionally, in Q we provide the proportion of vaccinated individuals by SES in Senario 1 (panel a) and Senario 2 (panel b).

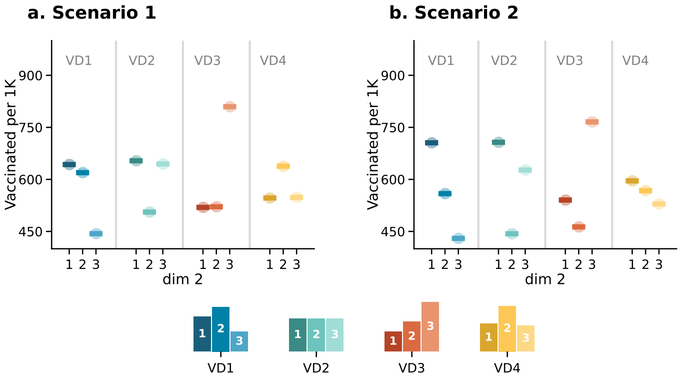

Figure Q: **Vaccination rates per 1,000 individuals by SES group under different vaccination distribution strategies using real-world contact data**. Panel a refers to Scenario 1 and panel b to Scenario 2. Results refer to the median of 500 runs with IQRs (shaded area). Epidemiological parameters: $\Gamma = 0.25, \Psi = 0.4$, $g_1 = 0.6$ and $g_2 = 0.8$. $R_0 = 2.7$ for Scenario 1 and $R_0 = 3$ for Scenario 2. Simulations start with $I_0 = 100$ initial initial infectious cases.

9.3 Epidemic outcomes using real-world contact matrices

For completeness, also in this case we also explore the number of deaths estimated by our model under the two different scenarios when the population distribution and the mixing patterns are taken from real data. Fig RA and Fig RC show the results for Scenario 1 while panels Fig RB and Fig RD refers to Scenario 2.

Additionally, here we present the same results stratified by age and vaccination status. These figures explore the dynamics of infection and mortality under different vaccination distribution scenarios ($VD1$, $VD2$, and $VD3$).

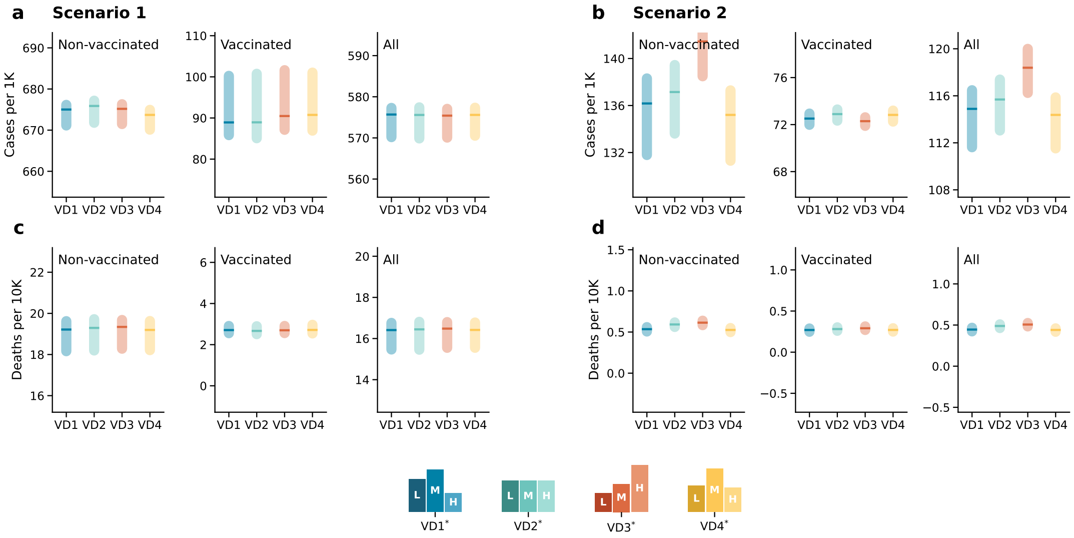

Figure R: **Epidemic outcomes under different vaccination distributions and real contact data.**. Panels in a and c refer to Scenario 1 and display the number of cases per 1000 (panel a) and the number of deaths per 10000 (panel c), stratified by vaccination status (non-vaccinated, vaccinated, and overall) and under different vaccination distribution scenarios ($VD1, VD2, VD3, VD4$). Panels in b and d show the corresponding number for Scenario 2. Results refer to the median of 500 runs with IQRs (shaded area). Epidemiological parameters: $\Gamma = 0.25, \Psi = 0.4$, $g_1 = 0.6$ and $g_2 = 0.8$. $R_0 = 2.7$ for the third wave and $R_0 = 3$ for the forth. Simulations start with $I_0 = 100$ initial infectious cases.

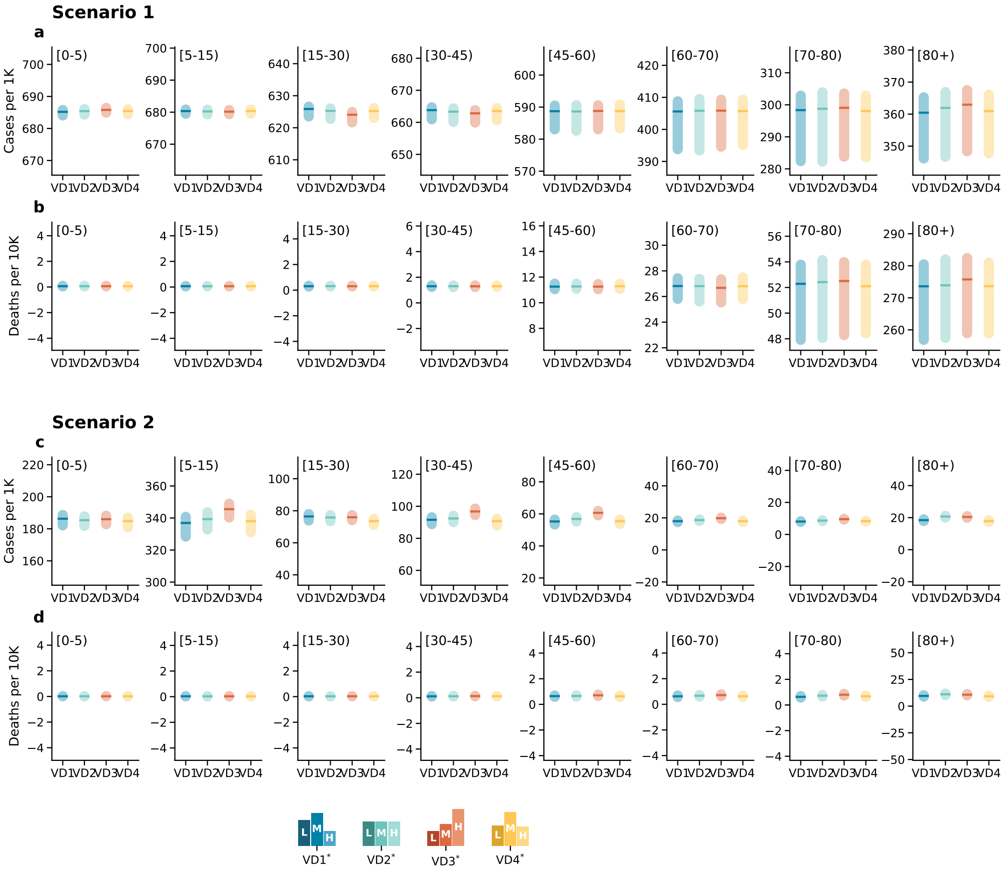

Figure S: **Epidemic outcomes under different vaccination distributions and real contact data, stratified by age for the entire population.** Panels (a) and (b) refer to Scenario 1. Panel (a) displays the number of cases per 1000 individuals under different vaccination distributions ($VD1, VD2, VD3, VD4$), while panel (b) shows the corresponding number of deaths per $10,000$ individuals. Panels (c) and (d) present the same results for Scenario 2. Results represent the median of 500 simulations with confidence intervals. Epidemiological parameters: $\Gamma = 0.25$, $\Psi = 0.4$, $g_1 = 0.6$, and $g_2 = 0.8$. $R_0 = 2.7$ for the third wave and $R_0 = 3$ for the fourth. Simulations start with $I_0 = 100$ initial infectious cases.

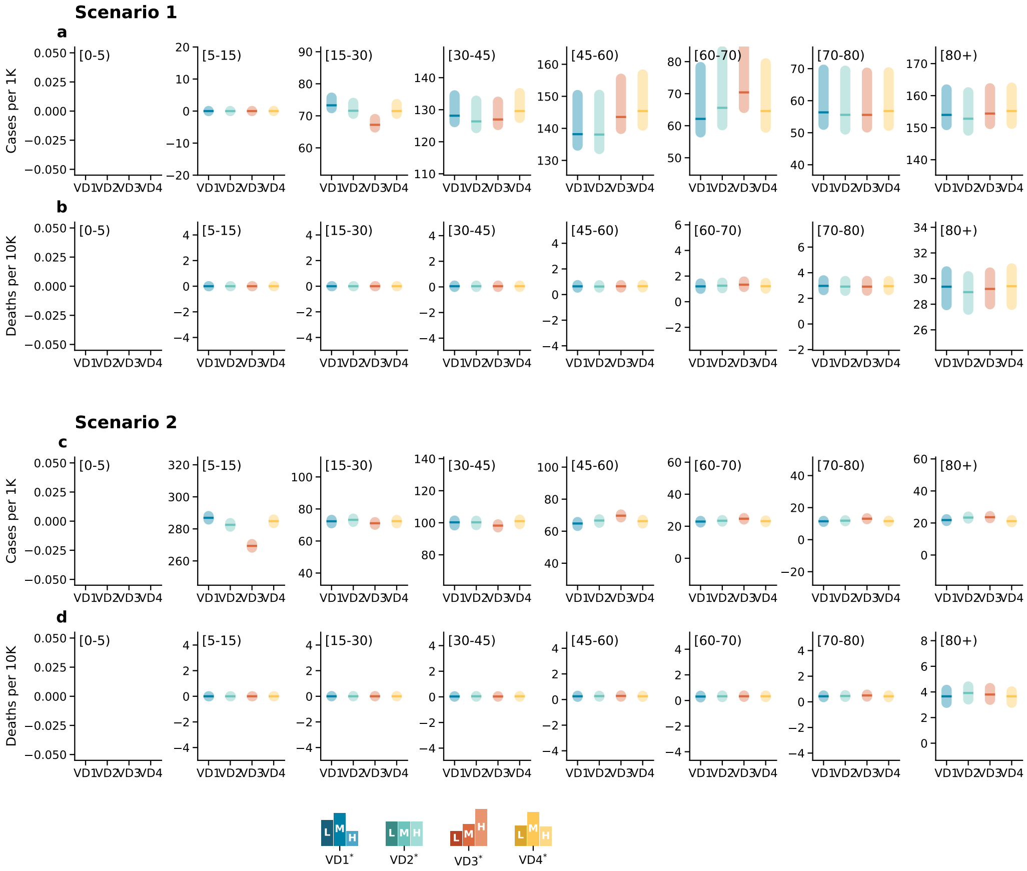

Figure T: **Epidemic outcomes under different vaccination distributions and real contact data, stratified by age for the vaccinated population.** Panels (a) and (b) refer to Scenario 1. Panel (a) displays the number of cases per 1000 individuals under different vaccination distributions ($VD1, VD2, VD3, VD4$), while panel (b) shows the corresponding number of deaths per $10,000$ individuals. Panels (c) and (d) present the same results forScenario 2. Results represent the median of 500 simulations with confidence intervals. Epidemiological parameters: $\Gamma = 0.25$, $\Psi = 0.4$, $g_1 = 0.6$, and $g_2 = 0.8$. $R_0 = 2.7$ for the third wave and $R_0 = 3$ for the fourth. Simulations start with $I_0 = 100$ initial infectious cases.

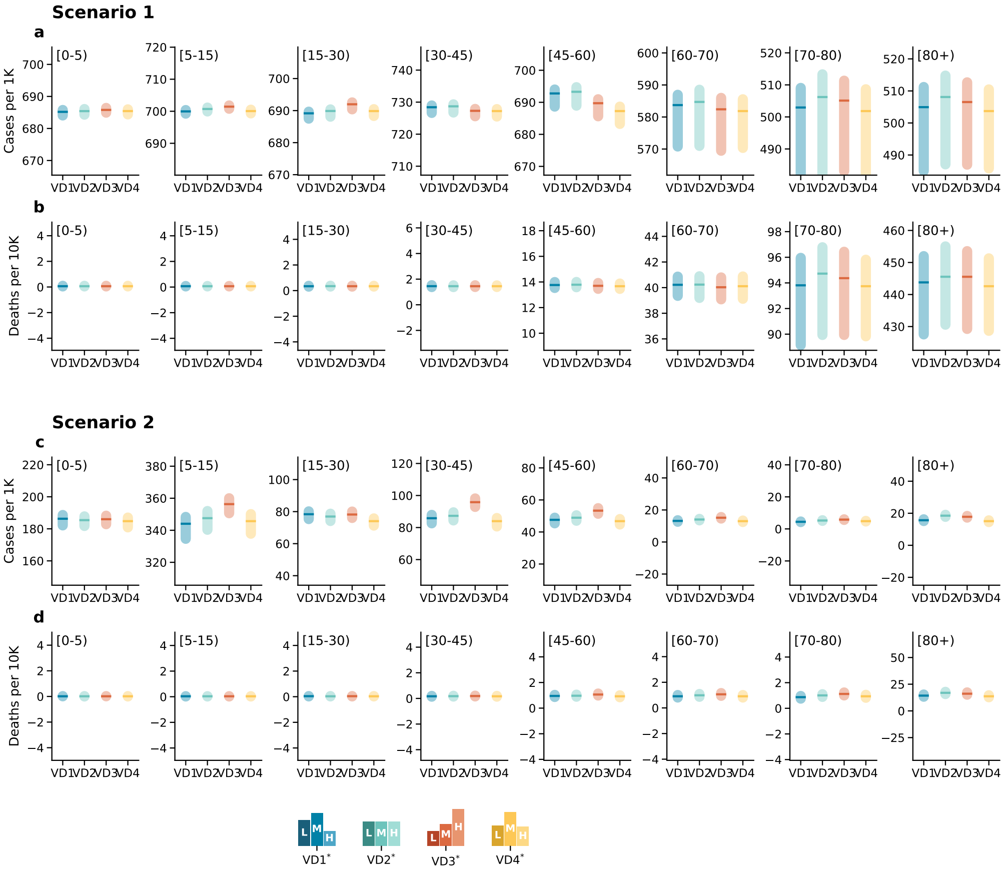

Figure U: **Epidemic outcomes under different vaccination distributions and real contact data, stratified by age for the non-vaccinated population.** Panels (a) and (b) refer to Scenario 1. Panel (a) displays the number of cases per 1000 individuals under different vaccination distributions $(VD1, VD2, VD3, VD4)$, while panel (b) shows the corresponding number of deaths per $10,000$ individuals. Panels (c) and (d) present the same results Scenario 2. Results refer to the median of 500 runs with IQRs (shaded area). Epidemiological parameters: $\Gamma = 0.25$, $\Psi = 0.4$, $g_1 = 0.6$, and $g_2 = 0.8$. $R_0 = 2.7$ for Scenario 1 and $R_0 = 3$ for Scenario 2. Simulations start with $I_0 = 100$ initial infectious cases.

References

[1] Our World in Data. Share of people who completed the initial covid-19 vaccination protocol by age, 2024. Accessed: 2024-07-10.

[2] Márton Karsai, Júlia Koltai, Orsolya Vásárhelyi, and Gergely Röst. Hungary in mask/maszk in Hungary. *Corvinus Journal of Sociology and Social Policy*, (2), 2020.

[3] Júlia Koltai, Orsolya Vásárhelyi, Gergely Röst, and Márton Karsai. Reconstructing social mixing patterns via weighted contact matrices from online and representative surveys. *Scientific Reports*, 12(1):1–12, 2022.

[4] Nemzeti adatvédelmi és információ szabadság hatóság, date of access 2023.05.23.

[5] Surveillance definitions for COVID-19, European Centre for Disease Prevention and Control, date of access 2023.05.23.

[6] Adriana Manna, Lorenzo Dall'Amico, Michele Tizzoni, Márton Karsai, and Nicola Perra. Generalized contact matrices allow integrating socioeconomic variables into epidemic models. *Science Advances*, 10(41):eadk4606, 2024.

[7] Joël Mossong, Niel Hens, Mark Jit, Philippe Beutels, Kari Auranen, Rafael Mikolajczyk, Marco Massari, Stefania Salmaso, Gianpaolo Scalia Tomba, Jacco Wallinga, et al. Social contacts and mixing patterns relevant to the spread of infectious diseases. *PLoS medicine*, (3):e74, 2008.

[8] Kiesha Prem, Alex R Cook, and Mark Jit. Projecting social contact matrices in 152 countries using contact surveys and demographic data. *PLoS computational biology*, 13(9):e1005697, 2017.

[9] Dina Mistry, Maria Litvinova, Ana Pastore y Piontti, Matteo Chinazzi, Laura Fumanelli, Marcelo FC Gomes, Syed A Haque, Quan-Hui Liu, Kunpeng Mu, Xinyue Xiong, et al. Inferring high-resolution human mixing patterns for disease modeling. *Nature communications*, 12(1):323, 2021.

[10] P Rohani M Keeling. *Modeling Infectious Diseases in Humans and Animals*. Princeton University Press, 2008.

[11] Henrik Salje, Cécile Tran Kiem, Noémie Lefrancq, Noémie Courtejoie, Paolo Bosetti, Juliette Paireau, Alessio Andronico, Nathanaël Hozé, Jehanne Richet, Claire-Lise Dubost, et al. Estimating the burden of sars-cov-2 in france. *Science*, 369(6500):208–211, 2020.

List of Figures

A **Hungary COVID-19 vaccination. a.** Share of people who completed the initial COVID-19 vaccination protocol by age **b.** Total Daily Dose for 100 people by age. . . . 3

B **Hungarian population distribution and age contact matrix:** (1*st row*) Population distribution by age (2*nd row*) Age-contact matrix referred to the pre-pandemic period for Hungary. . . . 4

C **Generalized contact matrix with two dimensions: age and *dim2*** 7

D **Scenario 1. Epidemic outcomes and predictability**: Panel *a* displays the number of newly infected individuals over time. The dotted black line corresponds to the outcome of the age-stratified model (i.e., $\mathbf{C}_{ij}$), and the solid coloured lines correspond to the outcome of the generalized model (i.e., $\mathbf{G_{ab}}$) in the three different vaccination strategies ($VD1, VD2, VD3$, $VD4$). Panel *b* shows the percentage of vaccinated individuals in the three subgroups (i.e., *dim2*). Panel *c* shows the attack rate by the second dimension predicted by the generalized model. Panel *d* shows the difference between the attack rate predicted by the generalized model and the one estimated from the aggregate output of the age-stratified model (i.e., $f(\mathbf{C}_{ij})$) by the second dimension. Results refer to the median of 500 runs with IQRs (shaded area). Epidemiological parameters: $\Gamma = 0.25, \Psi = 0.4$, $g_1 = 0.6$, $R_0 = 2$. Simulations start with $I_0 = 100$ initial infectious seeds. . . . 14

E **Scenario 2. Epidemic outcomes and predictability**: Panel *a* displays the number of newly infected individuals over time. The dotted black line corresponds to the outcome of the age-stratified model (i.e., $\mathbf{C}_{ij}$), and the solid coloured lines correspond to the outcome of the generalized model (i.e., $\mathbf{G_{ab}}$) in the three different vaccination strategies ($VD1, VD2, VD3, VD4$). Panel *b* shows the percentage of vaccinated individuals in the three subgroups (i.e., *dim2*). Panel *c* shows the attack rate by the second dimension predicted by the generalized model. Panel *d* shows the difference between the attack rate predicted by the generalized model and the one estimated from the aggregate output of the age-stratified model (i.e., $f(\mathbf{C}_{ij})$) by the second dimension. Results refer to the median of 500 runs with IQRs (shaded area). Epidemiological parameters: $\Gamma = 0.25, \Psi = 0.4$, $g_1 = 0.6$, $R_0 = 2$. Simulations start with $I_0 = 100$ initial infectious seeds. . . . 15

F **Scenario 1. Epidemic outcomes and predictability**: Panel *a* displays the number of newly infected individuals over time. The dotted black line corresponds to the outcome of the age-stratified model (i.e., $\mathbf{C}_{ij}$), and the solid coloured lines correspond to the outcome of the generalized model (i.e., $\mathbf{G_{ab}}$) in the three different vaccination strategies (i.e., $VD1, VD2, VD3, VD4$). Panel *b* shows the percentage of vaccinated individuals in the three subgroups (i.e., *dim2*). Panel *c* shows the attack rate by the second dimension predicted by the generalized model. Panel *d* shows the difference between the attack rate predicted by the generalized model and the one estimated from the aggregate output of the age-stratified model (i.e., $f(\mathbf{C}_{ij})$) by the second dimension. Results refer to the median of 500 runs with IQRs (shaded area). Epidemiological parameters: $\Gamma = 0.25, \Psi = 0.4$, $g_1 = 0.6$, $R_0 = 2$. Simulations start with $I_0 = 100$ initial infectious seeds. . . . 17

G **Scenario 2. Epidemic outcomes and predictability**: Panel *a* displays the number of newly infected individuals over time. The dotted black line corresponds to the outcome of the age-stratified model (i.e., $\mathbf{C}_{ij}$), and the solid colored lines correspond to the outcome of the generalized model (i.e., $\mathbf{G_{ab}}$) in the three different vaccination strategies ($VD1, VD2, VD3, VD4$). Panel *b* shows the percentage of vaccinated individuals in the three subgroups (i.e., *dim2*). Panel *c* shows the attack rate by the second dimension predicted by the generalized model. Panel *d* shows the difference between the attack rate predicted by the generalized model and the one estimated from the aggregate output of the age-stratified model (i.e., $f(\mathbf{C}_{ij})$) by the second dimension. Results refer to the median of 500 runs with IQRs (shaded area). Epidemiological parameters: $\Gamma = 0.25, \Psi = 0.4$, $g_1 = 0.6$, $R_0 = 2$. Simulations start with $I_0 = 100$ initial infectious seeds. . . . 17

H **Scenario 1. Epidemic outcomes and predictability**: Panel a displays the number of newly infected individuals over time. The dotted black line corresponds to the outcome of the age-stratified model (i.e., $\mathbf{C}_{ij}$), and the solid coloured lines correspond to the outcome of the generalized model (i.e., $\mathbf{G_{ab}}$) in the three different vaccination strategies (i.e., $VD1, VD2, VD3, VD4$). Panel b shows the percentage of vaccinated individuals in the three subgroups (i.e., *dim2*). Panel c shows the attack rate by the second dimension predicted by the generalized model. Panel d shows the difference between the attack rate predicted by the generalized model and the one estimated from the aggregate output of the age-stratified model (i.e., $f(\mathbf{C}_{ij})$) by the second dimension. Results refer to the median of 500 runs with IQRs (shaded area). Epidemiological parameters: $\Gamma = 0.25, \Psi = 0.4$, $g_1 = 0.6$, $R_0 = 2$. Simulations start with $I_0 = 100$ initial infectious seeds. 18

I **Scenario 2. Epidemic outcomes and predictability**: Panel a displays the number of newly infected individuals over time. The dotted black line corresponds to the outcome of the age-stratified model (i.e., $\mathbf{C}_{ij}$), and the solid colored lines correspond to the outcome of the generalized model (i.e., $\mathbf{G_{ab}}$) in the three different vaccination strategies (i.e., $VD1, VD2, VD3, VD4$). Panel b shows the percentage of vaccinated individuals in the three subgroups (i.e., *dim2*). Panel c shows the attack rate by the second dimension predicted by the generalized model. Panel d shows the difference between the attack rate predicted by the generalized model and the one estimated from the aggregate output of the age-stratified model (i.e., $f(\mathbf{C}_{ij})$) by the second dimension. Results refer to the median of 500 runs with IQRs (shaded area). Epidemiological parameters: $\Gamma = 0.25, \Psi = 0.4$, $g_1 = 0.6$, $R_0 = 2$. Simulations start with $I_0 = 100$ initial infectious seeds. 19

J **Sensitivity analysis to varying t_{epi} and R_0.** Differences between the overall attack rate for each subgroup as predicted by the generalized model $\mathbf{G}_{ab}$ and the attack rate predicted by the aggregate output of the age-stratified model, $f(\mathbf{C}_{ij})$ as a function of (i) t_{epi} with $R_0 = 2$, and (ii) R_0 with $t_{epi} = 0$. These analyses are done both under a random mixing setting (panel a) and an assortative mixing setting (panel b). Results refer to the median of 500 runs with IQRs (shaded area). 21

K **Sensitivity analysis to varying t_{epi} and R_0.** Differences between the overall attack rate for each subgroup as predicted by the generalized model $\mathbf{G}_{ab}$ and the attack rate predicted by the aggregate output of the age-stratified model, $f(\mathbf{C}_{ij})$ as a function of (i) t_{epi} with $R_0 = 2$, and (ii) R_0 with $t_{epi} = 0$. These analysis are done both under a random mixing setting (panel a) and an assortative mixing setting (panel b). Results refer to the median of 500 runs with IQRs (shaded area). 21

L **Scenario 1: The impact of NPIs on SES groups under different vaccination distributions.** Panel a refers to the baseline case and shows the attack rate scaled by 1000 for the three SES. Panel b follows the same structure but refers to the case where tightened NPIs are introduced, reducing contacts by 20%. NPIs are adjusted 65 days after the epidemic onset. Results represent the median of 500 runs with confidence intervals. Epidemiological parameters: $\Gamma = 0.25$, $\Psi = 0.4$, $g_1 = 0.6$, $g_2 = 0.8$, and $R_0 = 2$. Simulations begin with $I_0 = 100$ initial infected seeds. 23

M **Scenario 1: The impact of NPIs under different vaccination distributions.** Panel a refers to the baseline case, with insets depicting the activity distribution along the second dimension. The first column shows the total number of infected individuals, while the second column shows the attack rate scaled by 1000. Panel b follows the same structure but refers to the case where tightened NPIs are introduced, reducing contacts by 20%. Panels c and d follow the same structure but refer to the total number of deaths (first column) and mortality rate scaled by $10K$ (second column). NPIs are adjusted 65 days after the epidemic onset. Results refer to the median of 500 runs with IQRs (shaded area). Epidemiological parameters: $\Gamma = 0.25$, $\Psi = 0.4$, $g_1 = 0.6$, $g_2 = 0.8$, and $R_0 = 2$. Simulations begin with $I_0 = 100$ initial infected seeds. 23

N **Scenario 2: The impact of NPIs on SES groups under different vaccination distributions.** Panel a refers to the baseline case and shows the attack rate scaled by 1000 for the three SES. Panel b follows the same structure but refers to the case where tightened NPIs are released and the contacts increase by 20%. Panels c and d follow the same structure but refer to the total number of deaths (first column) and mortality rate scaled by $10K$ (second column). NPIs are adjusted 65 days after the epidemic onset. Results refer to the median of 500 runs with IQRs (shaded area). Epidemiological parameters: $\Gamma = 0.25$, $\Psi = 0.4$, $g_1 = 0.6$, $g_2 = 0.8$, and $R_0 = 2$. Simulations begin with $I_0 = 100$ initial infected seeds. 24

O **Scenario 2: The impact of NPIs under different vaccination distributions.** Panel a refers to the baseline case, with insets depicting the activity distribution along the second dimension. The first column shows the total number of infected individuals, while the second column shows the attack rate scaled by 1000. Panel b follows the same structure but refers to the case where tightened NPIs are released, increasing contacts by 20%. NPIs are adjusted 65 days after the epidemic onset. Results refer to the median of 500 runs with IQRs (shaded area). Epidemiological parameters: $\Gamma = 0.25$, $\Psi = 0.4$, $g_1 = 0.6$, $g_2 = 0.8$, and $R_0 = 2$. Simulations begin with $I_0 = 100$ initial infected seeds. . 24

P **Age-specific assortativity by socioeconomic status (SES)**. For each age group and SES level (low: L, medium: M, high: H), the index measures the proportion of contacts individuals have with others of the same SES group. Higher values indicate stronger assortative mixing by SES. Results are shown for Scenario 1 (left) and Scenario 2 (right), based on contact matrices stratified by age and SES. 25

Q **Vaccination rates per 1,000 individuals by SES group under different vaccination distribution strategies using real-world contact data**. Panel a refers to Scenario 1 and panel b to Scenario 2. Results refer to the median of 500 runs with IQRs (shaded area). Epidemiological parameters: $\Gamma = 0.25, \Psi = 0.4$, $g_1 = 0.6$ and $g_2 = 0.8$. $R_0 = 2.7$ for Scenario 1 and $R_0 = 3$ for Scenario 2. Simulations start with $I_0 = 100$ initial initial infectious cases. 26

R **Epidemic outcomes under different vaccination distributions and real contact data.**. Panels in a and c refer to Scenario 1 and display the number of cases per 1000 (panel a) and the number of deaths per 10000 (panel c), stratified by vaccination status (non-vaccinated, vaccinated, and overall) and under different vaccination distribution scenarios ($VD1, VD2, VD3, VD4$). Panels in b and d show the corresponding number for Scenario 2. Results refer to the median of 500 runs with IQRs (shaded area). Epidemiological parameters: $\Gamma = 0.25, \Psi = 0.4$, $g_1 = 0.6$ and $g_2 = 0.8$. $R_0 = 2.7$ for the third wave and $R_0 = 3$ for the forth. Simulations start with $I_0 = 100$ initial infectious cases. 27

S **Epidemic outcomes under different vaccination distributions and real contact data, stratified by age for the entire population.** Panels (a) and (b) refer to Scenario 1. Panel (a) displays the number of cases per 1000 individuals under different vaccination distributions ($VD1, VD2, VD3, VD4$), while panel (b) shows the corresponding number of deaths per $10,000$ individuals. Panels (c) and (d) present the same results for Scenario 2. Results represent the median of 500 simulations with confidence intervals. Epidemiological parameters: $\Gamma = 0.25$, $\Psi = 0.4$, $g_1 = 0.6$, and $g_2 = 0.8$. $R_0 = 2.7$ for the third wave and $R_0 = 3$ for the fourth. Simulations start with $I_0 = 100$ initial infectious cases. 28

T **Epidemic outcomes under different vaccination distributions and real contact data, stratified by age for the vaccinated population.** Panels (a) and (b) refer to Scenario 1. Panel (a) displays the number of cases per 1000 individuals under different vaccination distributions ($VD1, VD2, VD3, VD4$), while panel (b) shows the corresponding number of deaths per $10,000$ individuals. Panels (c) and (d) present the same results forScenario 2. Results represent the median of 500 simulations with confidence intervals. Epidemiological parameters: $\Gamma = 0.25$, $\Psi = 0.4$, $g_1 = 0.6$, and $g_2 = 0.8$. $R_0 = 2.7$ for the third wave and $R_0 = 3$ for the fourth. Simulations start with $I_0 = 100$ initial infectious cases. 29

U **Epidemic outcomes under different vaccination distributions and real contact data, stratified by age for the non-vaccinated population.** Panels (a) and (b) refer to Scenario 1. Panel (a) displays the number of cases per 1000 individuals under different vaccination distributions $(VD1, VD2, VD3, VD4)$, while panel (b) shows the corresponding number of deaths per $10,000$ individuals. Panels (c) and (d) present the same results Scenario 2. Results refer to the median of 500 runs with IQRs (shaded area). Epidemiological parameters: $\Gamma = 0.25$, $\Psi = 0.4$, $g_1 = 0.6$, and $g_2 = 0.8$. $R_0 = 2.7$ for Scenario 1 and $R_0 = 3$ for Scenario 2. Simulations start with $I_0 = 100$ initial infectious cases. 30

List of Tables

A List of parameters used to generate Fig 2 in the main text. 7
B Epidemiological parameters used in the SEIR model with vaccination. 8
C Vaccination distributions (VD) along the second dimension used in the simulations. . . . 10
D List of parameters used to simulate the NPIs 22
E Infection Fatality Rate by age group 22
